# Integrated multi-omics analysis reveals candidate genes for cuticular wax biosynthesis and molecular characteristics of a glossy mutant in rapeseed under natural drought stress

**DOI:** 10.3389/fpls.2026.1874927

**Published:** 2026-07-29

**Authors:** Lei Lei, Xianmin Meng, Weirong Wang, Hongwei Li, Jifeng Zhu

**Affiliations:** Key Laboratory of Germplasm Innovation and Genetic Improvement of Grain and Oil Crops (Co-construction by Ministry and Province), Ministry of Agriculture and Rural Affairs, Key Laboratory of Agricultural Genetics and Breeding of Shanghai, Crop Breeding and Cultivation Research Institute, Shanghai Academy of Agricultural Sciences, Shanghai, China

**Keywords:** *Brassica napus L.*, *CER1*, cuticular wax, drought, multi-omics analysis

## Abstract

**Introduction:**

Cuticular wax plays a crucial role in drought tolerance. However, the regulatory mechanisms controlling cuticular wax biosynthesis and the drought response in rapeseed are not well understood.

**Methods and Results:**

In this study, we identified a glossy mutant, *hy7201*, in rapeseed (*Brassica napus* L.). Compared to the wild-type ‘HY7201’, *hy7201* exhibited a significant reduction in total cuticular wax content, altered composition, and decreased crystal density, along with a significant increase in cuticle permeability. Genetic analysis revealed that the glossy phenotype of the *hy7201* mutant is controlled by a single dominant gene. Through bulked segregant analysis coupled with next-generation sequencing (BSA-seq) of waxy and glossy pools derived from F1 individuals of the self-crossed *hy7201* population, *BnaA09G0721400ZS*, the homolog gene of *AtCER1*, was identified as a key candidate gene. This gene encodes a very-long-chain aldehyde decarbonylase, which contributes to the differences in leaf cuticular wax accumulation between the wild-type ‘HY7201’ and the glossy mutant ‘*hy7201*’. To validate the key genes involved in cuticular wax biosynthesis and characterize the molecular features under natural drought conditions in glossy plants, we performed an integrated analysis of the leaf transcriptome, proteome, and metabolome using KEGG enrichment, Pearson correlation, and two-way orthogonal partial least squares (O2PLS) methods with three biological replicates. The genes CER1 (*BnaA09G0721400ZS*) and its paralog *CER1-2* (*BnaA09G0698500ZS*) were significantly downregulated at both the transcriptional and protein levels. Additionally, they exhibited significant negative correlations with differentially expressed metabolites (DEMs) in glossy plants under drought stress. The multi-omics approach uncovered that pathways related to cutin, suberin, and wax biosynthesis, as well as ABC transporters, glucosinolate biosynthesis, glutathione metabolism, linoleic acid metabolism, sphingolipid metabolism, and arginine and proline metabolism, significantly contribute to the glossy plant’s response to drought.

**Discussion:**

These results suggest that the drought response in the glossy mutant involves not only a defective cuticular barrier but also extensive metabolic and signaling reprogramming. This insight could facilitate the identification of genes related to cuticular wax biosynthesis and drought stress response, which could be utilized in molecular breeding programs to enhance drought tolerance in rapeseed. Consequently, this offers a promising approach for developing more sustainable farming methods amid environmental challenges.

## 1 Introduction

Plant cuticular wax is a hydrophobic lipid layer that coats the aerial organs of plants, such as leaves, fruit, flowers, and stems. This layer as the first line of defense in plants plays a crucial role in improving drought resistance by limiting water loss through non-stomatal routes (Lewandowska et al., 2020; Li et al., 2025a; Liu et al., 2025). Cuticular wax is mainly composed of very long-chain fatty acids (VLCFAs) and their derivatives with carbon chain lengths ranging from 20 to 48 carbons, and is synthesized in epidermal cells (Lee and Suh, 2013; Liu et al., 2026a; Pascal et al., 2019; Pu et al., 2013; Tomasi et al., 2024). Initially, C16 and C18 fatty acids are *de novo* synthesized in the plastid and subsequently elongated into VLCFAs, which serve as precursors for wax biosynthesis. This elongation process is mediated by the fatty acid elongase (FAE) complex, either independently or in coordination with ECERIFERUM2 (CER2), within the endoplasmic reticulum (ER). Ultimately, these VLCFAs undergo further modifications via two distinct pathways: the alcohol-forming pathway, which produces primary alcohols and wax esters, and the alkane-forming pathway, which yields aldehydes, alkanes, secondary alcohols, and ketones (Lee and Suh, 2022). The directional shunting of these wax precursors is tightly regulated by the SOH1–CER3–CER1 module in response to environmental conditions (Karaca-Bulut et al., 2025; Lewandowska et al., 2020; Li et al., 2025a; Lu et al., 2025). Therefore, the content and composition of cuticular wax exhibiting high variability in response to environmental stress (Lewandowska et al., 2020; Lu et al., 2025). The regulatory mechanisms governing cuticular wax biosynthesis and drought response have been extensively investigated in various species, including *Arabidopsis thaliana*, rice, and wheat (Lee and Suh, 2022; Li et al., 2026a; Liu et al., 2022; Shim et al., 2023). These mechanisms predominantly operate at the transcriptional level, involving various transcription factor families, such as MYB, AP2/EREBP, and AP2/DREB, that activate or repress cuticular wax biosynthesis genes in response to drought (Lee and Suh, 2022; Li et al., 2026a; Liu et al., 2022; Zhao et al., 2026). Nevertheless, only a few transcription factors, BnaC9.MYB46, BnaMYB52, and BnaC9.DEWAX1, have been reported to regulate cuticular wax biosynthesis and response to drought in rapeseed (Jin et al., 2025; Wang et al., 2023; Wu et al., 2026).

The glossy trait is predominantly associated with the absence of cuticular wax with bright green phenotype (Huang et al., 2025; Mo et al., 1995; Pu et al., 2013). Therefore, glossy mutants usually serve as ideal models for dissecting the molecular mechanism of cuticular wax biosynthetic and drought response (Huang et al., 2025; Qi et al., 2025; Liu et al., 2021). Genetic analysis indicates that the glossy phenotype is typically regulated by recessive genes. For example, the glossy mutant in *Brassica rapa* is controlled by a single recessive gene, such as *BrCER2*, *BrMYB31*, or *BrWAX3* (Huang et al., 2025; Qi et al., 2025; Yang et al., 2022a). In *Brassica oleracea*, the glossy mutants B156 and TL28–1 are also controlled by single recessive genes, *BoGL5* and *BoCER2*, respectively (Han et al., 2021; Ji et al., 2021). The eceriferum (cer) mutant, *Nilla Glossy*, in rapeseed exhibits a glossy trait governed by two recessive alleles, *BnaA1.CER4* and *BnaC1.CER4* (Liu et al., 2021). Nevertheless, only a limited number of reports have identified dominant glossy mutants in recent years. In wheat, the glaucousness trait is primarily regulated by two sets of dominant genes, *W1* and *W2* (Zhang et al., 2013). In banana, the nonwaxy pseudostem was controlled by a single dominant gene, *Wx* (Ortiz et al., 1995). In rapeseed, a dominant *glossy* mutant (*BnaA.GL*) and putative the *CER1* gene located on chromosome A09 was identified as the candidate gene (Pu et al., 2013). These glossy mutants often exhibited a dramatic reduction in total wax content, wax crystals on the leaf and stem surface, along with significantly increased cuticle permeability. These physiological and metabolic changes are closely related to the decline in drought tolerance. Although glossy mutants have been well investigated in model species such as *Arabidopsis thaliana* and cereal crops, the molecular mechanisms underlying cuticular wax biosynthesis and their regulatory networks under natural drought conditions remain largely unexplored in rapeseed (Daszkowska-Golec et al., 2023; McNevin et al., 1993; Roy et al., 2026; Tan et al., 2024; Zheng et al., 2021). In particular, multi-omic approach, encompassing transcriptomic, proteomic, and metabolomic profiling, has limited to be applied to characterize glossy mutants of rapeseed under realistic drought scenarios. This knowledge gap limits our understanding of species-specific drought responses and constrains the potential use of glossy traits in breeding programs.

Rapeseed, one of the most important oil crops worldwide, is highly susceptible to drought stress, which can cause significant yield losses (Hu et al., 2022a). In this study, we investigated a *glossy* mutant, ‘*hy7201’*, derived from the yellow-seed rapeseed cultivar ‘HY7201’ and discovered in the field, to identify candidate genes involved in cuticular wax biosynthesis and to elucidate the regulatory network under natural drought conditions. We conducted a comparative analysis of cuticle permeability, total cuticular wax content and composition between the leaves of the mutant *‘hy7201’* and the wild type ‘HY7201’. Subsequently, BSA-seq was employed to pinpoint candidate genes associated with cuticular wax biosynthesis. Furthermore, an integrative multi-mics approach combining transcriptomic, proteomic, and metabolomic analyses was utilized to uncover the underlying molecular mechanism of glossy rapeseed subjected to natural drought stress. We hypothesized that the glossy phenotype results from a single gene mutation affecting cuticular wax biosynthesis and that drought stress will amplify specific molecular signatures. The results may facilitate the identification of genes involved in cuticular wax biosynthesis and drought stress response, which can be utilized in molecular breeding programs designed to improve drought resistance in rapeseed, thereby offering a potential pathway toward more sustainable agricultural practices in the face of climate change.

## 2 Materials and methods

### 2.1 Plant materials and drought conditions

The glossy mutant ‘*hy7201*’, which derived from a natural mutation of the wild type waxy rapeseed materials ‘HY7201’, was founded in the field. The wild type ‘HY7201’, the glossy mutant ‘*hy7201*’ and F_1_ populations generated by the self-pollination of the mutant ‘*hy7201*’ all were cultivated under natural field conditions at the research base of the Shanghai Academy of Agricultural Sciences (30.88°N, 121.38°E, Shanghai, China). ‘HY7201’ is a high-quality yellow-seeded variety characterized by mosaic leaves, developed by the Shanghai Academy of Agricultural Sciences.

Leaves were collected under natural field conditions for the BSA-seq analysis on F_1_ mapping population generated by the self-pollination of the mutant ‘*hy7201*’. Multi-omics was performed on the same populations under natural drought conditions in the field resulting from seasonal climate changes. The natural drought conditions in this study refer to a moderate to severe drought stress levels, characterized by soil water content consistently below 50% (Zhang et al., 2023; Yin et al., 2025) for 17 days (November 14, 2025 to November 30, 2025), and the fluctuation range of soil moisture content in 1–28 cm layer was 33.94%–49.92%, the average soil water content was 40.57% (**Supplementary Figure 1A**). Additionally, the total rainfall during this period was 0.38 mm, and the fluctuation range of daily average temperature were 7.3°C–17.6 °C (**Supplementary Figure 1B**, S1C). The leaf samples for the transcriptome, proteome, and metabolome analyses were collected at 8-week-old seedlings of waxy (W) and glossy (G) plants from F_1_ population, with healthy leaves at the same leaf position. The samples for the three-omics analysis were from the same batch, and each leaf sample was pooled samples collected from five different rapeseed seedlings, and three biological repeats were used for each group (G_1, G_2, G_3, W_1, W_2, and W_3). Additionally, in order to enhance the reliability of the metabolic data, three additional biological replicates of each group (G_4, G_5, G_6, W_4, W_5, and W_6) were added specifically for the metabolome analysis, each replicate also comprising five individual leaves. All samples were harvested, stored, and processed uniformly to minimize environmental variations and ensure comparability.

### 2.2 Toluidine blue assay

Toluidine blue, a metachromatic dye, can attach to unbound anionic groups like carboxylate and phosphate. The TB assay assesses water permeability caused by cuticular wax defects on leaves surface, as described previously (Pu et al., 2013). Leaves of six-week-old seedling were soaked in a 0.05% (w/v) toluidine blue solution for 60 minutes and subsequently rinsed with water to remove excess TB from the leaf surface.

### 2.3 Measurement of leaf water loss rate

Leaves from six-week-old seedlings (HY7201 and *hy7201*) grown under normal field conditions were used to assess water loss. Initially, the leaves underwent a 12-hour dark treatment to ensure stomatal closure, after which their weight was recorded as the original mass (W1). Fresh weight (W2) was then measured every hour for up to 9 hours. The percentage of water loss was then calculated using the formula:


Water loss rate=[(W1−W2)/W1]×100%

### 2.4 Chlorophyll leaching assay

Chlorophyll leaching assay was performed as described by Tao et al. (2024). 2 cm segments were excised from the middle part of the second leaf of six-week-old seedlings from the WT ‘HY7201’ and ‘*hy7201*’, and immediately immersed in 5 mL 80% ethanol, and then kept in the dark. At time points of 0.5, 1, 1.5, 2, 2.5, 3, 4, 5, 6, 7, and 24 hours, absorbance at 647 nm and 664 nm was measured using a TECAN Infinite 200 PRO multimode reader in the order of samples. Chlorophyll content was calculated using the formula:


Chlorophyll content=7.93×A664+19.53×A647

The chlorophyll leaching rate was determined as follows:


Chlorophyll leaching rate=(chlorophyll content at each time point/chlorophyll content at 24 hours)×100%

Three replicates of each genotype were performed.

### 2.5 Scanning electron microscopy analysis

Leaves from the same position on 6-week-old rapeseed seedlings of ‘HY7201’ and ‘*hy7201*’ were used for scanning electron microscopy (SEM) observation. The dried leaf segments were sputter-coated with gold for 60 seconds using an SD-900 sputter coater (VPI, Beijing, China) and then examined with a TM4000Plus tabletop microscope (Hitachi, Tokyo, Japan).

### 2.6 Total content and composition analysis of leaf cuticular wax

The same position leaves located below the growth point of 6-week-old seedlings on wild type ‘HY7201’ and glossy mutant ‘*hy7201*’ were used for cuticular wax total content and composition analyses. Prior to cuticular wax extraction, the leaf was photographed adjacent to a sticker of known dimensions for surface area calculation using ImageJ software. Leaf sections (~20 cm^2^) were immersed into 10 mL chloroform with 10 μg n-tetracosane (C24) as an internal standard at 30 °C for 30 s and extracted for twice. The two supernatants were combined, dried under nitrogen, and weighed. Subsequently, the sample was transferred to a GC vial, to which equal volumes of pyridine and BSTFA (containing 1% TMCS, v/v) were added. The mixture was gently mixed well, and incubated in an oven at 70 °C for 45 minutes. Afterward, the derivatization reagent was dried with nitrogen and redissolve it with n-hexane. Finally, the solution was filtered through a 0.22 μm membrane before being loaded onto the machine.

After cooling, the derivatives were subject to GC-MS analysis using a fused-silica column TG-5MS (30 m×0.25 mm×0.25 μm) with helium as carrier gas at a flow rate of 1.2 mL/min, conducted on a TSQ 8000 EVO gas chromatography-tandem mass spectrometer system (70 eV, m/z 40 to 500) in constant velocity mode. The temperatures of the injector and MSD interface were both set to 250 °C while that of the ion source temperature was set to 230 °C. The GC analysis was conducted with an initial oven temperature of 40 °C for 1 minute, followed by a ramp at 60 °C/min to 280 °C, where it was held for 8 minutes, then increased at 10 °C/min to 320 °C, and maintained at 320 °C for 7 minutes. The conditions were provided by Nanjing Webiolotech Testing Technology Co., Ltd (China, Nanjing). The quantification of each compound was based on peak areas relative to the internal standard (ITSD) tetracosane. Statistical significance was analyzed using the T-test function in GraphPad Prism 10.6.0 software.

### 2.7 Bulk segregants analysis sequencing

To map the mutant genes in *‘hy7201*’, F_1_ mapping populations were generated from the self-cross of ‘*hy7201*’ and used for BSA-seq analysis. Waxy and glossy phenotypes of the F_1_ population were assessed based on leaf characteristics in the field. Twenty individuals exhibiting waxy and glossy phenotypes were selected from the F_1_ population, and their DNA were extracted and mixed in equal amounts to construct ‘W’(waxy) and ‘G’ (glossy) bulk pools. Genomic DNA was extracted from fresh leaves of the wild type ‘HY7201’, twenty waxy and twenty glossy plants using the CTAB method with a plant genomic DNA extraction kit (ZP309, ZOMANBIO, China). 0.5 μg of total DNA was taken from each of the WT and two bulk pools for library construction. Sequencing libraries were constructed using the TruSeq DNA PCR-free prep kit (Illumina, 20015963, USA). 2 x 150 bp double-end sequencing using NovaSeq sequencer 6000 (Illumina, Inc., San Diego, CA, USA).

The raw data is saved in paired-end FASTQ format and the quality of the sequencing data was assessed using Fastp software (v0.23.1) and visualized using the R package. The high-quality data obtained after filtering were aligned to the reference genome *Brassica napus* ‘ZS11’ v0 (https://yanglab.hzau.edu.cn/BnIR/germplasm_info?id=ZS11.v0) using the BWA (0.7.12-r1039) MEM model. Following use ‘MarkDuplicates’ in the Picard (1.107) software package to remove Duplicates. After base quality recalibration, the UnifiedGenotyper program in the GATK v3.8 package was used to identify all variant sites (SNPs and InDels) within genes located in the physical intervals of the major QTL (Zhu et al., 2015). The effects of the identified SNPs and InDels, including frameshift deletions, frameshift insertions, stop gains, stop losses, and synonymous mutations, were assessed using ANNOVAR (https://annovar.openbioinformatics.org/en/latest/) with a gene-based annotation model and default settings. The physical intervals of the major QTL were aligned to the reference genome *Brassica napus* ‘ZS11’ v0 to identify the corresponding annotated genes. Bulk segregant analysis was performed using the Euclidean distance (ED) algorithm (Hill et al., 2013) and the G’-value method (Magwene et al., 2011). SNP loci exhibiting genotype differences between the two mixed pools were utilized to statistically analyze the depth of each base in the different mixed pools, and the Euclidean Distance (ED) value for each locus was calculated. The ED values calculated using the formula:


$$\text{ED}=\sqrt{{\left(\text{Aglossy}-\text{Awaxy}\right)}^{2}+{\left(\text{Cglossy}-\text{Cwaxy}\right)}^{2}+{\left(\text{Gglossy}-\text{Gwaxy}\right)}^{2}+{\left(\text{Tglossy}-\text{Twaxy}\right)}^{2}}$$

(ATCG) glossy indicates the frequency of A, T, C, and G base in the glossy pool, where (ATCG) waxy represents the frequency of A, T, C, and G base in the waxy pool. To eliminate the background noise caused by ED values with small differences, calculate ED^4 (the 4th power of the ED value) and perform LoessFit fitting on ED^4. Select ‘the median +3×standard deviation’ as the threshold. For the Gprime analysis, the G’ value was calculated using the QTLseqr package in R language to screen SNPs, and then the G-statistical value analysis was carried out using the runGprimeAnalysis function in the QTLseqr package with WINDOW GPRIME as the window.

### 2.8 Gene cloning and sequence analysis of candidate genes

Two candidate genes, *BnaA09G0721400ZS* and *BnaA09G0714600ZS*, within the candidate interval on Chr.A09 (65.29-65.86 Mb) were screened and cloned. The RNA and genomic DNA were extracted from the leaves of the reference genome ‘ZS11’, the WT ‘HY7201’, and waxy and glossy plants that are no longer separated from the F_2_ population with Trizol Reagent (15596018CN, Invitrogen Life Technologies) and a plant genomic DNA extraction kit (ZOMANBIO, ZP309). Reverse transcription was performed with 1 μg of total RNA using a TransScript^®^ All-in-One First-Strand cDNA Synthesis Kit (AT341, TransGen, China) to obtain the template cDNA. Gene-specific primers were designed using DNAMAN 6.0 (Lynnon Biosoft, USA) and the sequences of primers were listed in **Supplementary Table 2**. A 2 kb fragment upstream of the ATG start codon of *BnaA09G0721400ZS* and *BnaA09G0714600ZS* were amplified from genomic DNA as the promoter region of genes. The PCR reaction mixture, with a total volume of 15 μL, consisted of 1 μL genomic DNA or cDNA, 0.5 μL each of forward and reverse primers, 7.5 μL of 2× high-fidelity enzyme mix (Vazyme, Nanjing, China), and 5.5 μL ddH_2_O. The PCR program included an initial step at 94 °C for 3 min, followed by 35 cycles of 94 °C for 30 s, 55 °C for 30 s, and 72 °C for 150 s, 72 °C for 5 min, and a final hold at 4 °C. The PCR products were separated by 1% (w/v) agarose gel electrophoresis and conducted sanger sequencing by Sangon Biotech (Shanghai, China). The gene sequences were aligned using ESPript 3.2 (Robert et al., 2025).

### 2.9 RNA sequencing analysis

Total RNA was isolated using Trizol Reagent (15596018CN, Invitrogen Life Technologies), with 3 μg of RNA were used as input material for RNA sample preparations. Library preparation and high-throughput sequencing were carried out by Shanghai Personal Biotechnology Co. Ltd. The sequencing using the Illumina platform NovaSeq 6000. Raw sequencing data were used fastp (0.22.0) software to filter the sequencing data to obtain high quality sequence (clean data) for further analysis (Chen et al., 2018). Then, the filtered reads were mapping to the reference genome ZS11.v0 using HISAT2 (v2.1.0) (Kim et al., 2019; Song et al., 2020). And using HTSeq (v0.9.1) statistics to compare the read count values on each gene as the original expression of the gene (Anders et al., 2015). The differential expression analysis between the two comparison groups was analyzed by DESeq (v1.38.3) (Love et al., 2014) with screening criteria for DEGs in a |log2FoldChange|>1 and *p*-value< 0.05. Using topGO (v2.50.0) to perform GO enrichment analysis on differentially expressed genes (all DEGs/upregulated DEGs/downregulated DEGs), calculate *p*-value by hypergeometric distribution method (the standard of significant enrichment is *p*-value<0.05) (Alexa and Rahnenfuhrer, 2009). ClusterProfiler (v4.6.0) software was used to carry out the KEGG enrichment pathway analysis of differential expression genes, focusing on the significant enrichment pathway with *p*-value<0.05 (Wu et al., 2021).

### 2.10 Quantitative real-time PCR analysis

Trizol Reagent (15596018CN, Invitrogen Life Technologies) was used to extract leaves total RNA from the waxy (W) and glossy (G) pools according to the manufacturer’s instructions. Reverse transcription was performed with 1 μg of total RNA using a TransScript^®^ All-in-One First-Strand cDNA Synthesis Kit (AT341, TransGen, China). Gene-specific primers were designed using the Primer-BLAST tool in National Center for Biotechnology Information (NCBI) (https://ncbi.nlm.nih.gov/tools/primer-blast/index.cgi), and the sequences of primers were listed in **Supplementary Table 1**. RT-qPCR was performed using the Perfect Start Green qPCR Super Mix Kit (AQ601-04, TransGen, China) on a QuantStudioTM 6 and 7 Flex Real-Time PCR System (Thermo Fisher Scientific, USA). Gene expression levels were calculated using the comparative Ct method with *BnaACTIN7* (*BnaC02G0037200ZS*) as the reference gene for data normalization (Liu et al., 2021). Three biological replicates were performed for each gene with three technical replicates per experiment. qRT-PCR reaction system consists of template cDNA (0.8 μL), specific primers (1.0 μL), SYBR qPCR Super Mix (7.5 μL), supplemented with nuclease-free water to a final volume of 15 μL.

### 2.11 Proteome analysis

Protein extraction involved fully grinding samples stored at −80 °C into powder using liquid nitrogen. Subsequently, 4 volumes of phenol extraction buffer containing 1% protease inhibitor were added to each sample, followed by ultrasonic lysis. An equal volume of Tris-balanced phenol was then added, and the mixture was centrifuged at 5500×g for 10 min at 4 °C. The supernatant was collected, mixed with 5 volumes of 0.1 M ammonium acetate/methanol solution, and incubated overnight for protein precipitation. The precipitates were sequentially washed with methanol and acetone. Finally, the pellets were redissolved in 1% SDS solution. The total protein content was determined using the BCA quantification kit (P0012, Beyotime). 15 μg of protein per sample was separated by 12% SDS−PAGE and stained with Coomassie Brilliant Blue to evaluate the quality and integrity of the protein samples. Equal amounts of protein from each sample were taken for digestion. The volume of each sample was adjusted to the same level with lysis buffer, and 4 volumes of acetone were added. After vortex mixing, proteins were precipitated overnight at -20 °C, followed by washing with acetone three times. The washed protein pellets were resuspended and supplemented with DTT to a final concentration of 5 mM, then incubated at 37 °C for 1 h for reduction. Subsequently, IAA was added to a final concentration of 11 mM, and the mixture was incubated at room temperature in the dark for 45 min for alkylation. Finally, trypsin was added to the alkylated proteins, and enzymatic digestion was performed overnight. The peptide samples were desalted using C18 desalting columns, which were sequentially activated with 100% acetonitrile (ACN) and equilibrated with 0.1% formic acid (FA). The peptide mixtures were loaded onto the columns, washed with 0.1% FA solution, and eluted with elution buffer (70% ACN, 0.1% FA). The eluates were collected, dried in a vacuum centrifugal concentrator, and stored at −20°C until further analysis.

Chromatographic separation for DIA analysis was performed using a nano-flow Vanquish Neo system (Thermo Fisher Scientific). After nano-scale high-performance liquid chromatography separation, the samples were subjected to data-independent acquisition (DIA) mass spectrometry with an Astral high-resolution mass spectrometer (Thermo Fisher Scientific). The detection mode adopted data-independent scanning (DIA). The precursor ion scanning range was set at 380–980 m/z. The full MS resolution was 240,000 at 200 m/z, with a normalized AGC target of 500% and a maximum injection time of 5 ms. For MS² acquisition, the DIA mode was applied with 300 variable isolation windows and an isolation window width of 2 m/z. The HCD collision energy was set to 25 eV. The normalized AGC target was 500%, and the maximum injection time was 3 ms. The rapeseed protein sequence database (personal_*Brassica_napus*_id3708_107233s_2025_09_11. fasta) was selected for protein identification analysis of mass spectrometry data and DIA-NN 1.9.2 was used for data analysis (Demichev et al., 2020). Differential protein analysis was conducted using the T-test, with fold changes >1.2 and *p*-value<0.05.

### 2.12 Metabolome analysis

Transfer 40 mg of the ground plant sample to a 2 mL centrifuge tube, then add 300 μL of pre-cooled methanol: acetonitrile: water (2:2:1, v/v/v, containing 5 ppm 2- chlorophenylalanine), along with 2 steel balls, and vortex for 30 s. Proceed by placing the mixture in a high-throughput tissue homogenizer, homogenizing at 55 Hz for 60 s, repeating this step once. Subsequently, subject it to ultrasonic cleaning for 10 minutes, followed by freezing in a -20 °C freezer for 30 minutes. Afterward, centrifuge at 12,000 rpm at 4 °C for 10 min, collect the supernatant, filter it through a 0.22 μm filter membrane, and transfer the filtrate to the test bottle. Take 10–20 μL of each sample filtrate and mix them to form a QC sample for evaluating instrument stability and data reliability.

Metabolites were separated using a ThermoVanquish Flex chromatograph (Thermo Fisher Scientific, Waltham, MA, USA) and identified in both positive and negative ion modes using a Thermo Orbitrap Exploris 120 mass spectrometer (Thermo Fisher Scientific, Waltham, MA, USA) equipped with a HESI source. An ACQUITY UPLC HSS T3 column (100Å, 1.8 µm, 2.1 mm × 100 mm) was used at a flow rate of 0.4 mL/min, a column temperature of 40 °C, an autosampler at 8 °C, and an injection volume of 2 μL. The mobile phases consisted of 0.1% formic acid in water (mobile phase A) and acetonitrile (containing 0.1% formic acid, v/v) (mobile phase B). The mass spectrometer parameters were as follows: spray voltage 3.5 kV/-3.0 kV, sheath gas 40 arb, auxiliary gas 10 arb, capillary temperature 320 °C, auxiliary gas temperature 300 °C, primary resolution 60,000, scan range 70–1000 m/z, AGC Target Standard, Max IT 100 ms, screening of the top 4 ions for secondary fragmentation, dynamic exclusion time 4 s, secondary resolution 15,000, HCD collision energy 30%, AGC Target Standard, Max IT Auto.

The raw data were imported into MS-DIAL software (version 4.9.221218) for subsequent analysis (Tsugawa et al., 2015). This software facilitated peak extraction, alignment, filtering, and metabolite identification. Compounds with more than 50% deletion within each group are filtered out, and undetected peaks are filled with missing values and normalized. Metabolite identification was utilized the PerSonalbio Next-Generation Metabolomics Database (PSNGM Database), which includes a self-built standard library, the mzCloud library (https://www.mzcloud.org/), LIPID MAPS (https://www.lipidmaps.org/), HMDB (https://hmdb.ca/), MoNA (https://mona.fiehnlab.ucdavis.edu/), NIST_2020_MSMS, and an AI-predicted MSMS map library. The primary search parameters included an MS1 tolerance for identification of 0.01, an MS2 tolerance for identification of 0.05, a smoothing level of 3, a minimum peak height of 10,000, a minimum peak width of 5, a mass slice width of 0.05, and an identification score cut-off of 70. Differential metabolites were screened by integrating three criteria: fold changes>1, *p*-value<0.05, and variable importance in projection (VIP) >1.

### 2.13 Multi-omics joint analysis

The multi-omics joint analysis is carried out in two steps. Firstly, the joint analysis of proteomics and transcriptomics is conducted, and then the combined analysis of the common DEGs/DEPs with the metabolome is performed. For the integrated analysis of proteomics and transcriptomics, we first obtain the quantitative detection and analysis results of proteomics and transcriptomics, and extract the proteins and transcripts with corresponding relationships. Then extract the differentially expressed proteins and the corresponding differentially expressed transcript relationship pairs to demonstrate the consistency of the relationship pairs. Then map the proteins and related transcripts to the relevant metabolic pathways. DEGs and DEPs between comparison groups were identified were used to conduct the Pearson correlation analysis based on the DEGs threshold line is |fold change (FC)| >2 and *p*-value<0.05, where the DEPs threshold line is |fold change (FC)| >1.2 and *p*-value<0.05. Finally, GO and KEGG enrichment analyses were conducted on the genes corresponding to the relationship pairs by topGO and clusterProfiler (V4.6.0) software.

Subsequently, the common DEGs/DEPs were further combining with metabolome data for joint analysis. Information on metabolites and transcripts was obtained respectively through two databases, KEGG Small molecules database (https://www.kegg.jp/kegg/compound/) and KEGG Orthology database (https://www.kegg.jp/kegg/ko.html) to acquire the information on differential metabolites and corresponding transcripts. And then conduct Pearson correlation analysis and O2PLS analysis to obtained correlation between DEMs and corresponding transcripts. The Pearson correlation coefficients of corresponding genes and metabolites were calculated by using the cor program in R package. The correlation between the total genes and metabolites expressed in each differential group is presented through a nine-quadrant chart based on the following criteria: |fold change (FC)| >2, VIP>1, and *p*-value<0.05. The O2PLS analysis was carried out by OmicsPLS package (Bouhaddani et al, 2018). Finally, the common pathways of the two omics difference enrichment analyses were sorted out by using clusterProfiler (V4.6.0) software, as well as the common annotation pathways of the differential metabolites and genes.

## 3 Results

### 3.1 Phenotypic comparison, total content and composition of cuticular waxes between the wild type ‘HY7201’ and the glossy mutant ‘*hy7201*’

**Figure 1:**
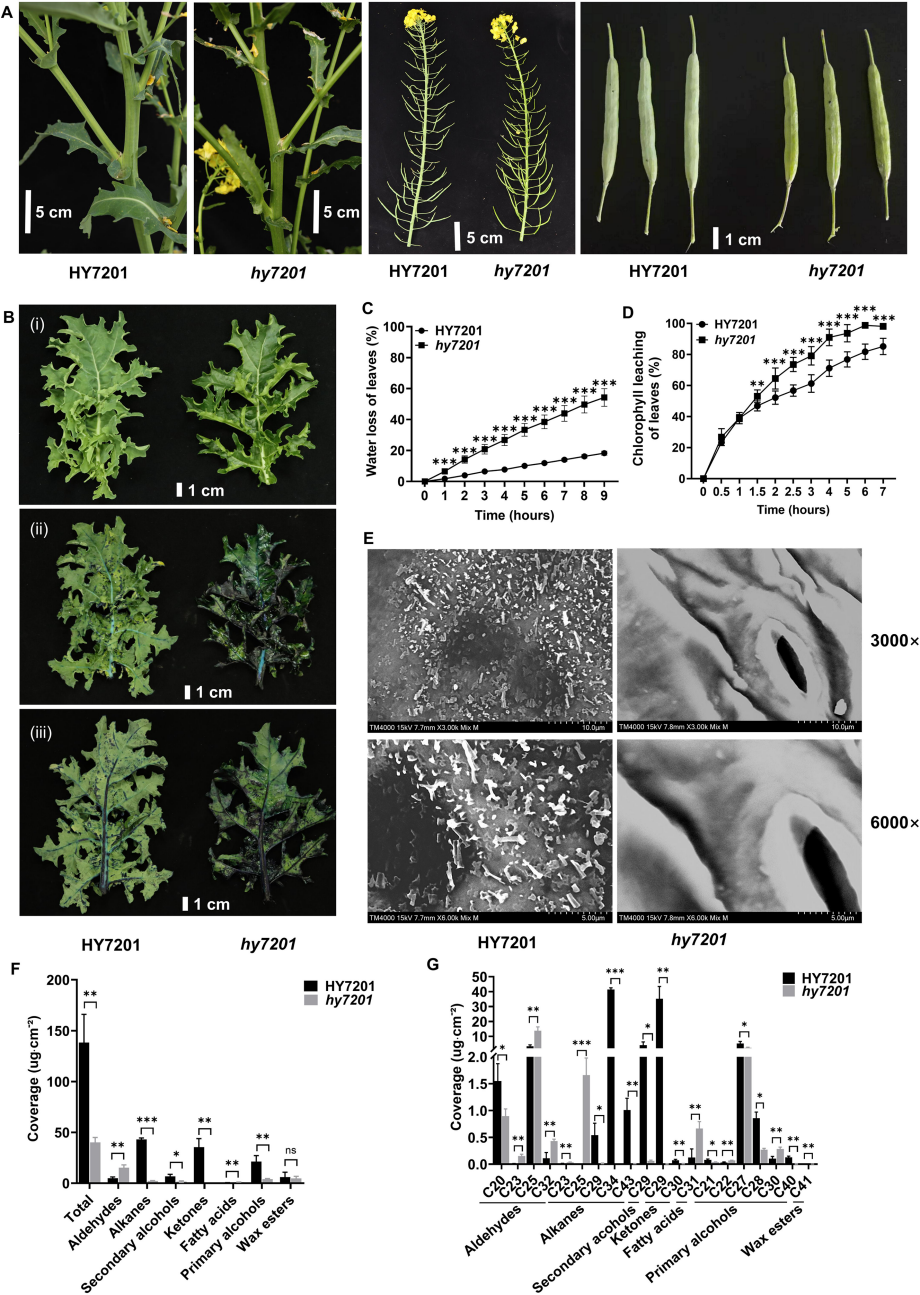
Phenotype and cuticular wax measurement of the WT ‘HY7201’ and the glossy mutant ‘*hy7201*’. **(A)** Comparison of the glaucousness phenotype of stems, inflorescence stems, and siliques between the WT (HY7201) and the glossy mutant (*hy7201*). **(B)** Glaucousness phenotype of leaves **(i)** and toluidine blue staining patterns of the WT and the glossy mutant on the front (B-ii) and back (B-iii) of the leaf. **(C)** Water loss rate of the WT and the glossy mutant leaves. **(D)** Chlorophyll leaching of the WT and the glossy mutant leaves. **(E)** Wax crystals on the leaf surface at magnifications of 3000× and 6000× of the WT and the glossy mutant with scanning electron micrographs. **(F)** Total content and components of cuticular wax on ‘HY7201’ and ‘*hy7201*’ leaves. **(G)** The main differentiating substances among the seven main components of cuticular wax on ‘HY7201’ and ‘*hy7201*’ leaves. The values in **(C, D, F, G)** represent the mean ± SD from three independent replicates. Statistical significance between the WT and the glossy mutant was determined using Student’s t-test, with asterisks ‘*’, ‘**’, and ‘***’ indicating significance at *p<* 0.05, *p* < 0.01, and *p* < 0.001, respectively.

A glossy green rapeseed mutant phenotype was identified within the breeding population of the wild type (WT) cultivar ‘HY7201’ under natural field conditions. Compared to the WT, the glossy mutant ‘*hy7201’* displayed a marked reduction glaucousness on the surfaces of leaves, stems, inflorescence stems and siliques (**Figures 1A, B**). This pronounced decrease in glaucousness on the leaves of the glossy mutant ‘*hy7201*’ corresponded with increased cuticle permeability, as evidenced by intense toluidine blue (TB) staining, significantly elevated rates of water loss, and enhanced chlorophyll leaching (**Figures 1B–D**). Concurrently, the glossy mutant ‘*hy7201*’ exhibited a marked reduction in the density of wax crystals on the leaf surfaces (**Figure 1E**). Furthermore, the amount and composition of cuticular wax on the leaves were analyzed using gas chromatography-mass spectrometry (GC-MS). The glossy mutant ‘*hy7201*’ exhibited a total wax coverage of 40.27 μg/cm^2^, representing a 70.9% reduction relative to the WT’HY7201’, which had a wax coverage of 138.25 μg/cm^2^ (**Figure 1F**). A total of 299 chemical metabolites, classified into nine categories, were identified within the cuticular waxes of both genotypes (**Supplementary Table 3**, **Excel S1**). No significant differences were observed in the categories, quantities, or proportions of compounds between the WT and the glossy mutant (**Supplementary Table 3**). Focused on the seven primary components of cuticular wax, which revealed that the levels of alkanes, secondary alcohols, ketones, and primary alcohols were significantly decreased in ‘*hy7201*’ (**Figure 1F**). In contrast, the contents of aldehydes and fatty acids were significantly increased in ‘*hy7201*’. No significant differences were observed in the wax ester content (**Figure 1F**). More specifically, significant increases were found in C25 aldehydes and C31 fatty acids, whereas significant reductions were observed in C29, C34, and C43 alkanes, C29 secondary alcohols, C29 and C30 ketones, C27 and C28 primary alcohols in ‘*hy7201*’ (**Figure 1G**). Notably, C29, C34, and C43 alkanes, C29 secondary alcohols, and C29 and C30 ketones were significantly reduced in the glossy mutant, particularly the alkanes (C29, C34) and ketones (C29, C30), which are the predominant components of total wax in rapeseed leaves (Pu et al., 2013; Tassone et al., 2016; Jin et al., 2020). Fatty acids and aldehydes, which as the initial products of VLCFAs modification in the alkane-forming pathway, were accumulated in ‘*hy7201*’, whereas the alkanes, secondary alcohols, and ketones, of subsequent products in the alkane-forming pathway were notably diminished. Therefore, these findings demonstrate that the mutations in the glossy mutant ‘*hy7201*’ primarily affect the conversion of aldehydes to alkanes in the alkane-forming pathway, resulting in reduced total wax content, altered composition, decreased crystal density, and increased cuticle permeability. Consequently, these physiological and metabolites changes in the glossy mutant likely contribute directly to its drought sensitivity.

### 3.2 BSA-seq identified *BnaA09G0721400ZS* (*CER1*) as the candidate gene controlling glossy phenotype

**Figure 2:**
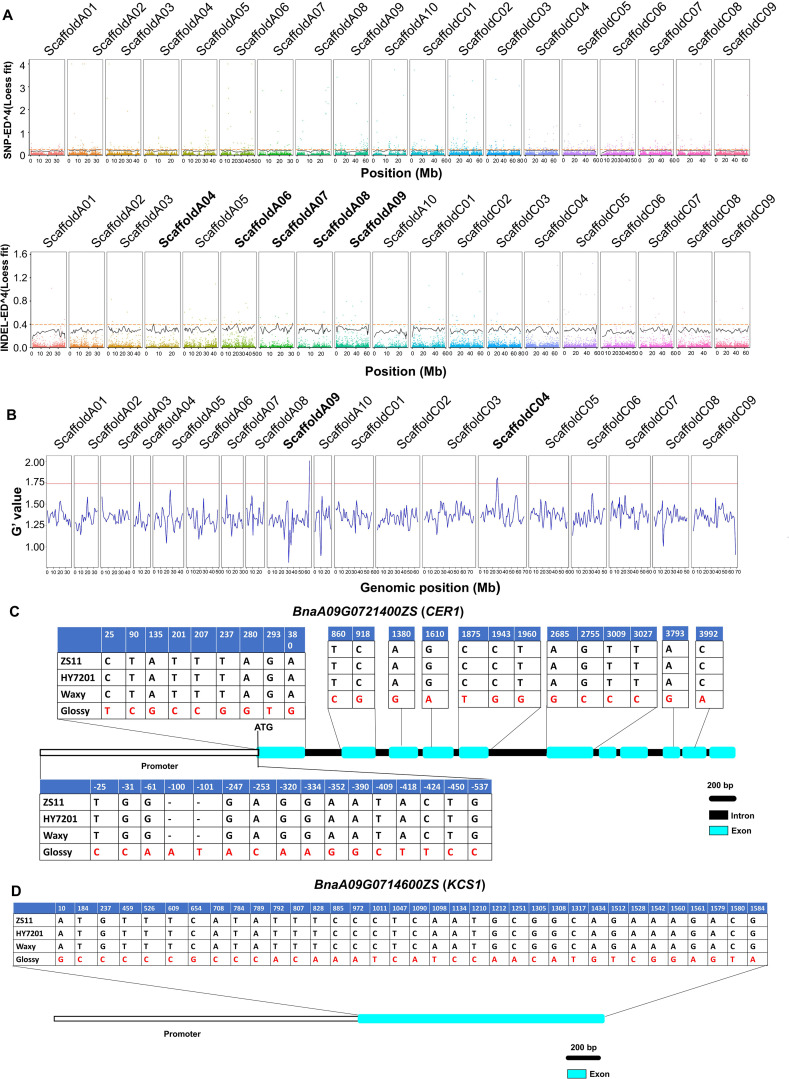
Manhattan plot illustrating the variation tends of ED and G’ value between the G-pool and W-pool associated with cuticular wax distribution across different chromosomes. **(A)** SNP-ED^4 and Indel-ED^4 between the G-pool and W-pool. The black line represents the fitted line, and the orange line indicates the threshold. **(B)** G’ value between the G-pool and W-pool. The blue line represents fitted line, and the red line indicates the threshold. **(C)** Variation sites in the promoter and coding sequence (CDS) regions of *BnaA09G0721400ZS* in glossy plants. **(D)** Variation sites in the coding sequence (CDS) regions of *BnaA09G0714600ZS* in glossy plants.

To investigate the genetic basis of the glossy mutant ‘*hy7201*’, we evaluated the leaf waxy phenotypes in F_1_ generation derived from the self-crossing of the mutant ‘*hy7201*’. The F_1_ individuals displayed a phenotypic ratio of glossy to waxy of 3:1 (glossy: waxy=116:38, χ^2^ = 0.0087<3.841). This result suggested that ‘*hy7201*’ is a dominant mutant with the glossy phenotype controlled by a single dominant gene. To further explore the candidate gene associated with the glossy phenotype, BSA-seq was conducted to identify the major QTL and candidate genes involved in cuticular wax biosynthesis. The ED and G ‘value methods, which possess higher mapping positional accuracy, were employed to analyze genetic mapping of cuticular waxy traits (Shen et al., 2019; Li and Xu, 2022). The results of ED method indicated that the candidate regions located on ChrA04, ChrA06, ChrA07, ChrA08 and ChrA09 (**Figure 2A**, **Table 1**). Subsequently, the G ‘value method was conduct to analyze the BSA-Seq data, identifying two candidate regions on ChrA09 and ChrC04. Surprisingly, the ED and G prime methods exhibit an overlapping region on the ChrA09 at 65.29-65.86 Mb (**Table 1**). Notably, the site on ChrA09 exhibited a higher and sharper the peak well above the threshold line in G ‘value method, suggesting a greater likelihood of the region on the ChrA09 at 65.29-65.86 Mb being the main effect region and other sites likely represent modifiers or noise (**Figure 2B**, **Table 1**).

**Table 1:** Candidate region associated with cuticular wax biosynthesis.

Analytical method	Chrom	Start	End
ED	ChrA09	65298276	65862011
	ChrA04	5634138	5814159
	ChrA06	19567934	22083182
	ChrA06	48513711	48704103
	ChrA07	16509885	17418500
	ChrA07	31912690	32244760
	ChrA08	20603908	20995950
Gprime	ChrA09	65247288	65862011
	ChrC04	26729972	28085784

Within this region, a total of 33 candidate genes were identified (Excel S1). Notably, among these genes, *BnaA09G0714600ZS* and *BnaA09G0721400ZS* stand out as they encode 3-ketoacyl-CoA synthase 1-like (KCS1) and a very-long-chain aldehyde decarbonylase (CER1), respectively, both playing roles in cuticular wax synthesis. Meanwhile, sequence analysis revealed that 16 and 22 SNP variations occurred in the promoter and CDS regions, respectively, of *BnaA09G0721400ZS* in glossy plants (**Figure 2C**, **S2**-**S4**), while 35 SNP variations were observed in the CDS region of *BnaA09G0714600ZS* (**Figure 2D**, **S6**, **S7**). The mutations in *BnaA09G0721400ZS* of glossy plants caused frameshift mutations in the first exon, resulting in a predicted protein product consisting of only an 8-amino acid sequence (**Supplementary Figure 5**). The mutations in *BnaA09G0714600ZS* of glossy plants led to four amino acid substitutions (**Supplementary Figure 8**). These mutations of *BnaCER1* and *BnaKCS1* are likely to result in alterations to gene function and expression. In addition, previous research has shown that CER1 predominantly produces C29 n-alkanes (89% of total hydrocarbons) alongside minor quantities of C25, C27, and C31 n-alkanes (Stéphanie et al., 2019). Meanwhile, the loss-of-function mutations of *CER1* block the conversion of stem wax C30 aldehydes (triacontanal) to C29 alkanes (nonacosane), and the secondary alcohols (C29) and ketones (C29 and C30) were also reduced in the mutants (Aarts et al., 1995; Bourdenx et al., 2011). On the other hand, KCSs are part of the FAE complex responsible for synthesizing very-long-chain fatty acids with specific chain lengths. In our current investigation, we observed a significant reduction in alkanes (C29, C34) and ketones (C29, C30) in the glossy mutant ‘*hy7201*’, while there was a notable increase in fatty acids, particularly C31, in ‘*hy7201*’. Due to the glossy phenotype being determined by a single dominant gene, and the component analysis of cuticular wax in the glossy mutant ‘*hy7201*’ indicating that the step converting aldehydes to alkanes was blocked, these findings suggest that *BnaA09G0721400ZS* is a promising candidate gene involved in the regulation of cuticular wax biosynthesis in rapeseed leaves and the mutation in *BnaA09G0721400ZS* directly resulting in the wax deficiency.

### 3.3 Transcriptome analysis and differential expression gene identification under natural drought conditions

**Figure 3:**
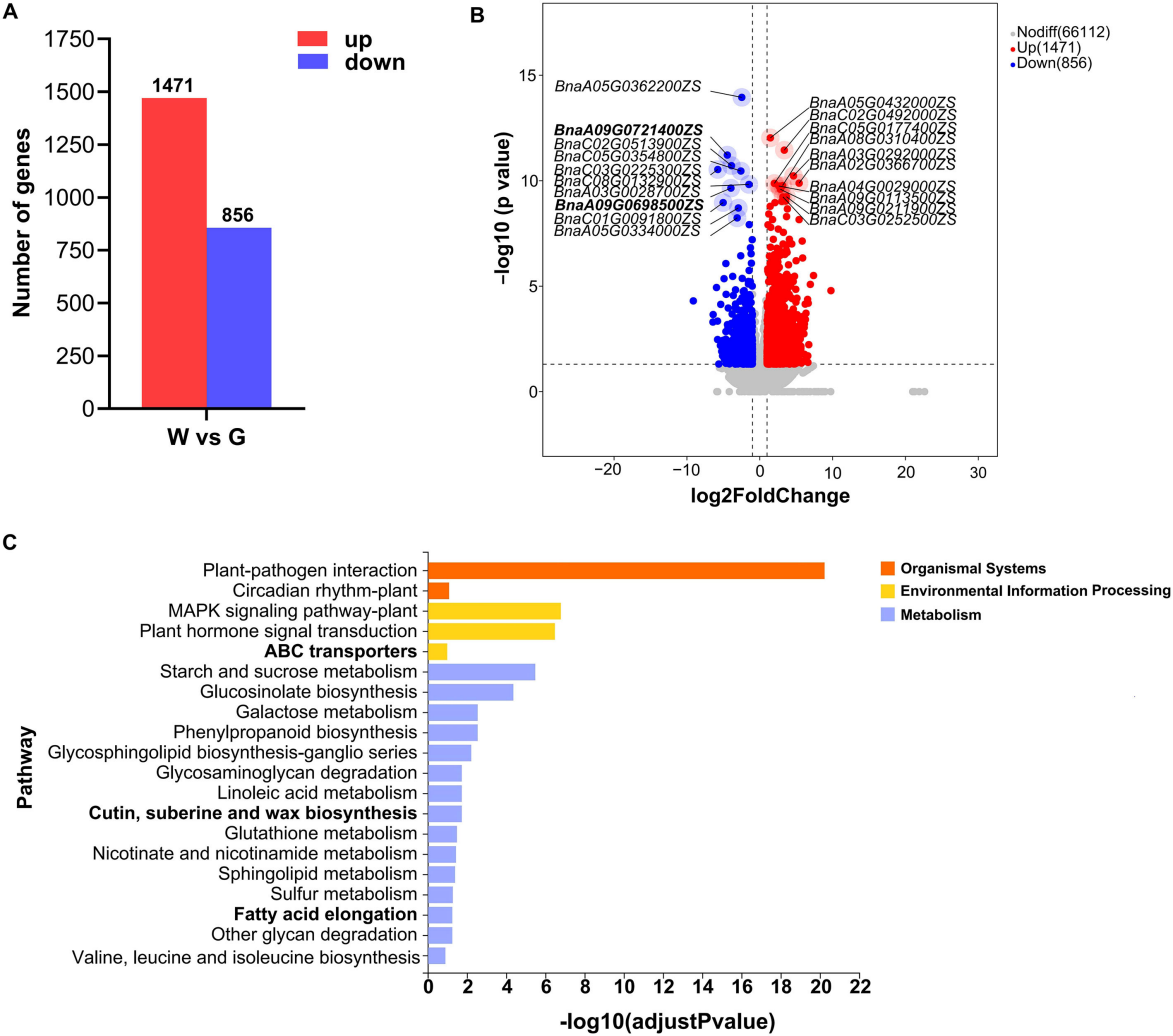
Transcriptional analysis of leaves from waxy and glossy plants under natural drought conditions. **(A)** Upregulated and downregulated differentially expressed genes (DEGs) between waxy (W) and glossy (G) plants. **(B)** The top ten most significantly upregulated and downregulated DEGs between waxy (W) and glossy (G) plants. **(C)** KEGG pathway analysis of 2,327 DEGs between waxy (W) and glossy (G) plants.

A total of 2,327 annotated unigenes exhibited differential expression, with 1,471 up-regulated and 856 down-regulated differentially expressed genes (DEGs) in the glossy (G) pool compared to the waxy pool under drought stress (**Figure 3A**). Notably, the candidate gene *CER1* (*BnaA09G0721400ZS*), identified through BSA-seq, was significantly downregulated in glossy plants. Additionally, its paralog *CER1-2* (*BnaA09G0698500ZS*) also exhibited significant downregulation (**Figure 3B**). However, the candidate gene *BnaA09G0714600ZS* (*KCS1L*), also identified by BSA-seq, did not show significant changes in glossy plants. *BnaA09G0721400ZS* previously as the candidate gene associated with cuticular wax synthesis also significantly decreased in *GL* mutant, and overexpression of *BnaCER1–2* has been demonstrated to enhance drought tolerance by promoting the accumulation of alkanes (C27, C28, C29, and C31) (Pu et al., 2013; Wang et al., 2020, 2023). These results confirm *CER1* (*BnaA09G0721400ZS*) as the candidate gene for controlling the dominant glossy phenotype. Gene Ontology (GO) enrichment analysis of 2,327 DEGs illustrated that the top 20 GO terms enriched in biological processes, including responses to lipid, hormones, and stress (**Supplementary Figure 9**). Among the 1,471 upregulated DEGs, significant enrichment was observed in processes related to responses to external stimuli and hormones (**Supplementary Figure 10A**). In contrast, the 856 downregulated DEGs were significantly enriched in process associated with cutin biosynthesis, secondary metabolite biosynthetic and metabolic, and the formation of plant-type cell walls (**Supplementary Figure 10B**). The KEGG enrichment analysis of the 2,327 DEGs revealed that the top 20 pathways were predominantly related to metabolism and environmental information processing, including MAPK signaling pathway, plant hormone signal transduction, ABC transporters, starch and sucrose metabolism, glucosinolate biosynthesis, galactose metabolism, phenylpropanoid biosynthesis, linoleic acid metabolism, cutin, suberin, and wax biosynthesis, glutathione metabolism, sphingolipid metabolism, and fatty acid elongation (**Figure 3C**). And the 1,471 up-regulated DEGs were predominantly enriched in MAPK signaling pathway, plant hormone signal transduction, and ABC transporters (**Supplementary Figure 10C**), where the 856 down-regulated DEGs exhibited significant enrichment in pathways including glucosinolate biosynthesis, phenylpropanoid biosynthesis, and linoleic acid metabolism (**Supplementary Figure 10D**).

**Figure 4:**
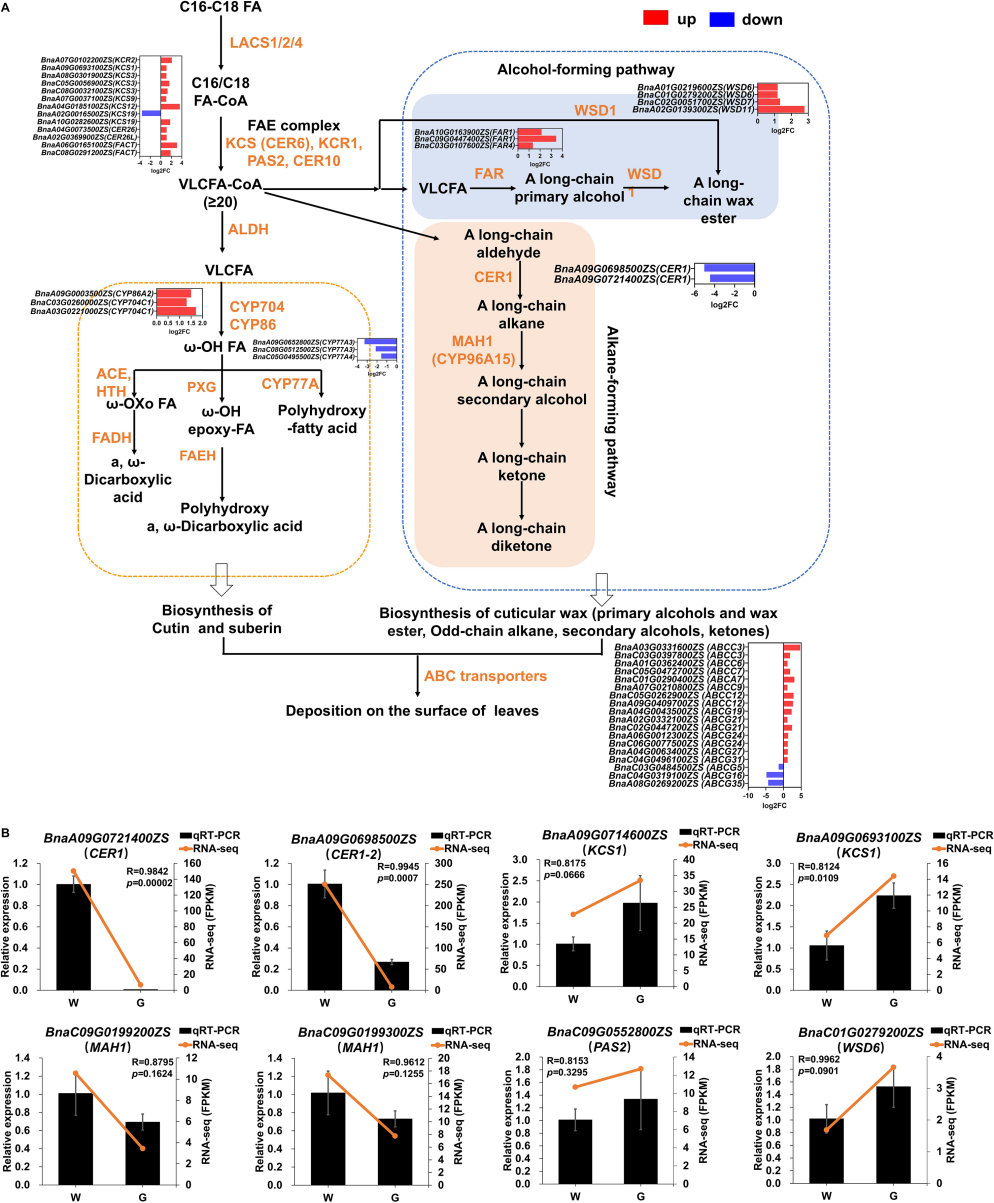
Transcriptional regulation of the cuticular wax synthesis and transport pathway in glossy plants under natural drought conditions. **(A)** DEGs in the cuticular wax synthesis and transport pathway under drought conditions. FA, fatty acid; VLCFC, very long-chain fatty acids. **(B)** qRT-PCR validation of transcription data. The black bar represents qRT-PCR, and the orange lines indicates RNA-seq values. The values represent the mean ± SD from three independent replicates. The R value represents the correlation between the two data sets. The *p*-value indicates the statistical significance of the relative expression levels between waxy (W) and glossy (G) plants, as analyzed by Student’s t-test.

Notably, 36 upregulated and 9 downregulated DEGs involved in cutin, suberin, and wax biosynthesis and transport in glossy plants under natural drought conditions (**Figure 4A**). In the alkane-forming pathway of wax synthesis, the *CER1* (*BnaA09G0721400ZS*) and its paralog *CER1-2* (*BnaA09G0698500ZS*) were significantly decreased by 4.456-fold and 5.029-fold, respectively (**Figure 4A**). In contrast, DEGs encode the fatty acyl reductases (FARs) (BnaC09G0447400ZS, BnaA10G0163900ZS, BnaC03G0107600ZS) and wax synthases (WSDs) (BnaA01G0219600ZS, BnaC01G0279200ZS, BnaC02G0051700ZS, BnaA02G0139300ZS) in the alcohol-forming pathway were all up-regulated (**Figure 4A**). Additionally, most genes encode components of FAE complex associating with fatty acid elongation and ABC transporter were up-regulated in glossy plants under drought (**Figure 4A**). Eight genes in cuticular wax synthesis and transport pathways were randomly selected for qRT-PCR analysis. The qRT-PCR results exhibited highly consistent expression trends with the RNA-seq data, with a strong positive correlation coefficient (R> 0.8) between the two datasets (**Figure 4B**), indicating the reliability of our transcriptomic profiling data. Furthermore, the genes encoding CYP86A2 and CYP704 in cutin and suberin biosynthesis were also significantly upregulated, whereas the genes *CYP77A* were significantly downregulated in glossy plants under drought. It appears that the downregulation of *CER1* and *CER1–2* gene expression in the alkane-forming pathway has led to the activation of DEGs in the alcohol-forming pathway and the cutin biosynthesis pathway in glossy plants under drought conditions (**Figure 4A**).

**Figure 5:**
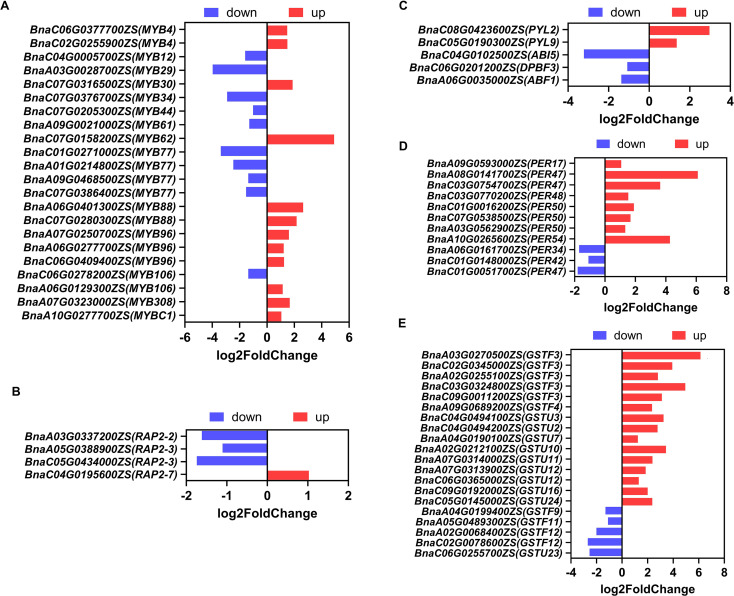
Differentially expressed genes (DEGs) involved in cuticular wax biosynthesis and drought response in glossy plants. **(A, B)** Key transcription factors regulating cuticular wax biosynthesis under drought conditions. **(C)** DEGs associated with the abscisic acid (ABA) signaling pathway. **(D)** DEGs related to antioxidant activity enriched in the phenylpropanoid biosynthesis pathway. **(E)** DEGs related to antioxidant activity enriched in glutathione metabolism pathways.

Moreover, due to glossy plants are more sensitive to drought stress, a series of DEGs related to stress response are significantly upregulated, including genes involved in abscisic acid (ABA) signaling and reactive oxygen species (ROS) scavenging (**Figure 5**). Previous research has highlighted the role of transcription factors such as MYB and AP2/DREB in regulating cuticular wax biosynthesis and response to drought stress by directly activating genes involved in cuticular wax biosynthesis (Lee et al., 2016; Yang et al., 2020; Jin et al., 2025; Li et al., 2026a; Li et al., 2026b; Zhao et al., 2026). In the current study, numerous DEGs encoding MYB were found to be up-regulated in glossy plants. For instance, *BnaC07G0316500ZS* encodes MYB30, and three genes (*BnaC06G0409400ZS*, *BnaA06G0277700ZS*, *BnaA07G0250700ZS*) encode MYB96, all showing significant up-regulation (**Figure 5A**). Conversely, DEGs encoding AP2/DREB-type transcription factors were down-regulated in glossy plants under natural drought conditions (**Figure 5B**). In addition, we observed a significant up-regulation of DEGs encoding the ABA receptors PYL2 and PYL9 in glossy plants under drought conditions, suggesting an elevation in ABA levels. However, three genes encoding ABI5, a downstream transcription factor of ABA, were notably down-regulated in glossy plants (**Figure 5C**). Additionally, a majority of DEGs enriched in the phenylpropanoid biosynthesis and glutathione metabolism pathways, involved in ROS scavenging, were up-regulated in glossy plants (**Figures 5D, E**). These findings imply that the increased vulnerability of glossy plants to drought stress is influenced not only by genes regulating cuticular wax synthesis but also by stress-related genes.

### 3.4 Proteome analysis and differentially expressed proteins identification under natural drought conditions

**Figure 6:**
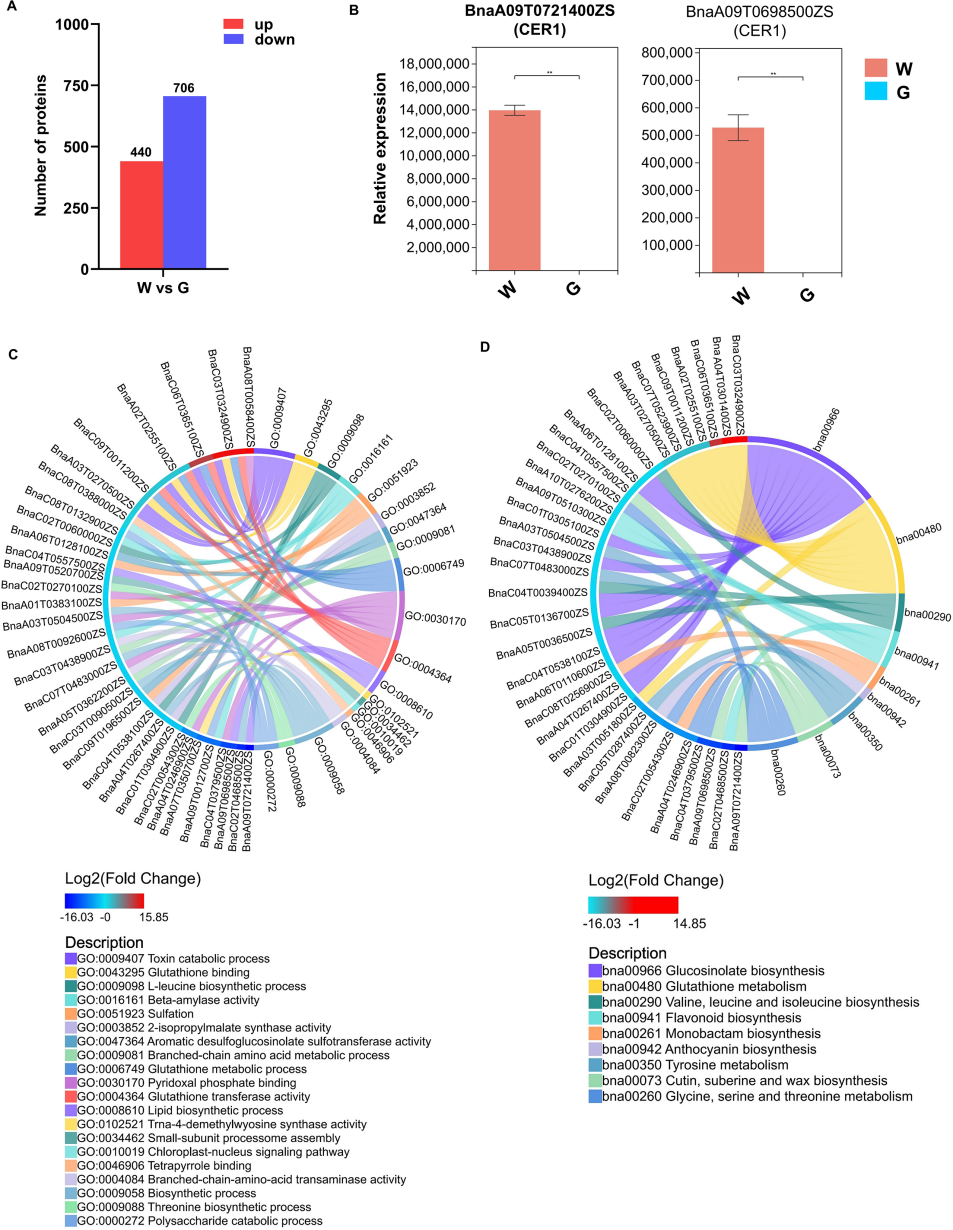
Characterization of differentially expressed proteins (DEPs) between waxy and glossy plants under natural drought conditions. **(A)** Histogram of upregulated and downregulated DEPs between waxy and glossy plants under natural drought conditions. **(B)** Relative expression of the candidate protein CER1 (BnaA09T0721400ZS and BnaA09T0698500ZS) in waxy and glossy plants under natural drought conditions. The values represent the mean ± SD from three independent replicates. Statistical significance was analyzed using Student’s t-test, with asterisks ‘**’ indicating significance at *p* < 0.01. **(C)** Gene Ontology (GO) term analysis of all DEPs between waxy and glossy plants under natural drought conditions. **(D)** KEGG pathway analysis of DEPs between waxy and glossy plants under natural drought conditions.

To elucidate the mechanisms of cuticular wax metabolism in the leaves of glossy rapeseed exposed to drought, we compared the relative protein expression between waxy (W) and glossy (G) plants. The data-independent acquisition (DIA) yielded 97,514 peptides and 13,910 proteins, of which 13,683 were quantified. Notably, 440 differentially expressed proteins (DEPs) were upregulated, while 706 DEPs were downregulated in glossy plants (|FC|>1.2 and *p* value< 0.05) (**Figure 6A**, **Excel S1**). Among all DEPs, the CER1, BnaA09G0721400ZS and BnaA09T0698500ZS, were significantly downregulated by 16.03 times and 11.31 times in glossy plants ((**Figure 6B**). And BnaA09G0721400ZS ranked among the top 40 most significantly altered differential proteins (**Supplementary Figure 11**). GO term analysis revealed that 1,146 DEPs significantly enriched in the lipid biosynthetic process (GO:0008610), glutathione binding (GO:0043295), glutathione metabolic process (GO:0006749), and glutathione transferase activity (GO:0004364) (**Figure 6C**). GO term analysis of 440 up-regulated DEPs showed that significantly enriched in glutathione binding, glutathione metabolic process, and glutathione transferase activity (**Supplementary Figure 12A**), while 706 down-regulated DEPs were enriched in L-leucine biosynthetic process (GO:0009098), beta-amylase activity (GO:0016161), and lipid biosynthetic process (GO:0008610) (**Supplementary Figure 12B**). Interestingly, BnaA09G0698500ZS, BnaA09G0721400ZS, and BnaC04G0557500ZS were enriched in the lipid biosynthetic process and all three proteins were annotated as CER1 or CER1-like proteins (**Figure 6C**, **S5B**). KEGG pathway analysis revealed that the 1,146 DEPs were enriched in pathways related to glucosinolate biosynthesis (bna00966), glutathione metabolism (bna00480), flavonoid biosynthesis (bna00941), anthocyanin biosynthesis (bna00942), and cutin, suberin and wax biosynthesis (bna00073) (**Figure 6D**). Seven DEPs enriched in the glutathione metabolism pathway were significantly upregulated in glossy plants, whereas DEPs associated with glucosinolate metabolism, flavonoid biosynthesis, anthocyanin biosynthesis, and cutin, suberin and wax biosynthesis were downregulated in glossy plants (**Figure 6D**, **S4C, D**). Correspondingly, 1146 DEPs were significantly enriched in domains such as very-long-chain aldehyde decarbonylase CER1-like (IPR021940), glutathione S-transferase (IPR010987, IPR004046, IPR004045, IPR036282), glycoside hydrolase (IPR001371, IPR018238) (**Supplementary Figure 13**). These results indicate a high degree consistency between DEPs and DEGs related to cuticular wax synthesis and antioxidant system pathways at both the protein and transcriptional levels.

### 3.5 Identification and functional annotation of differential metabolites

**Figure 7:**
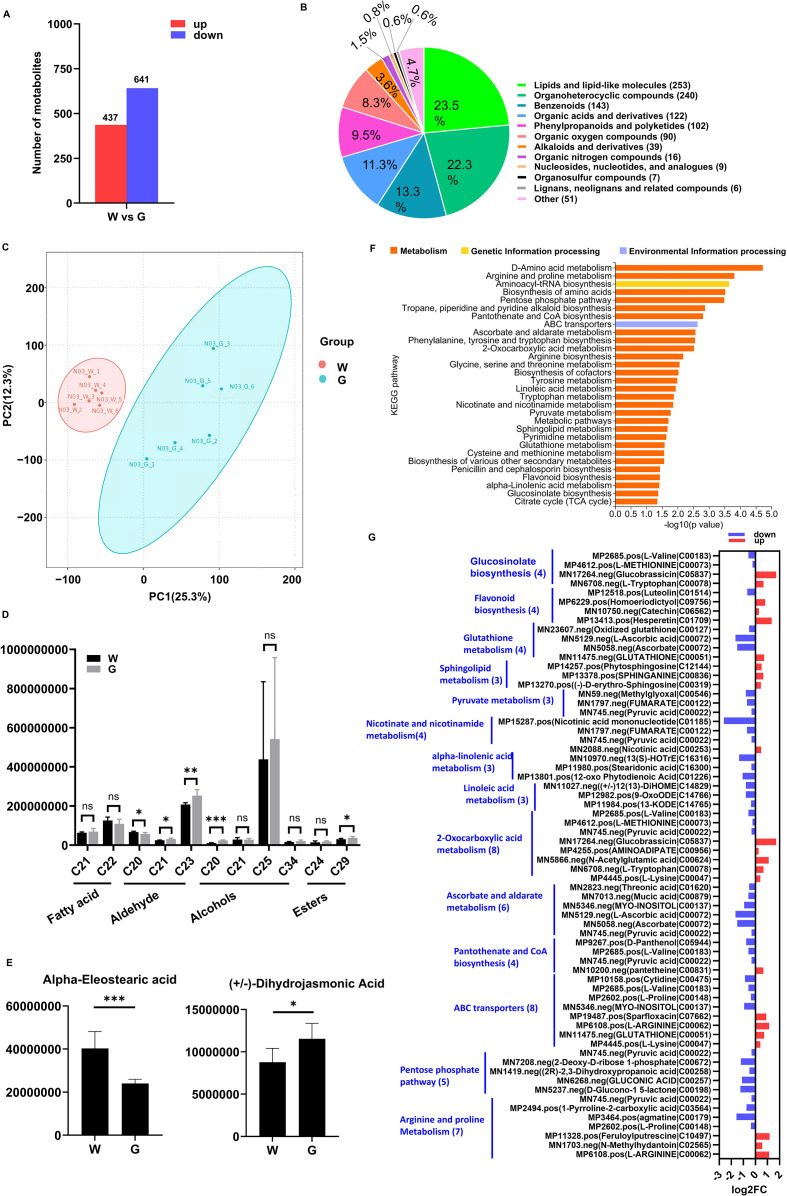
Metabolomic analysis of waxy and glossy plants under natural drought conditions. **(A)** Bar chart showing the number of upregulated and downregulated differentially expressed metabolites (DEMs) between the waxy and glossy plants under natural drought condition. **(B)** Pie chart illustrating the classification of 1,078 DEMs. **(C)** Principal component analysis of waxy and glossy samples. **(D)** VLCFAs and derivatives in waxy and glossy leaves under the drought. **(E)** Modulation of fatty acid metabolism and plant hormone signal transduction under drought stress. **(F)** KEGG pathway enrichment analysis of DEMs. **(G)** Histogram of depicting the upregulated and downregulated DEMs involved in KEGG pathways under natural drought. The values in **(D, E)** represent the mean ± SD from six independent replicates of quantitative results of metabolites. Statistical significance between waxy and glossy plants was analyzed using Student’s t-test, with asterisks ‘*’, ‘**’, and ‘***’ indicating significance at *p<* 0.05, *p* < 0.01, and *p<* 0.001, respectively.

To analyze the metabolite changes between waxy and glossy plants under natural drought conditions, we performed an untargeted metabolomics analysis using LC-MS/MS. A total of 1,078 differentially accumulated metabolites (DEMs) were identified in glossy plants, comprising 437 upregulated and 641 downregulated DEMs compared to waxy plants (**Figure 7A**, **Excel S1**). This finding suggests that glossy plants exhibited a greater number of downregulated genes than their waxy counterparts during natural drought. The 1,078 DEMs classified into 12 categories based on primary classification, including Lipids and lipid-like molecules (253), Organoheterocyclic compounds (240), Benzenoids (143), Organic acids and derivatives (122), Phenylpropanoids and polyketides (102), Organic oxygen compounds (90), Alkaloids and derivatives (39), Organic nitrogen compounds (16), Nucleosides, nucleotides, and analogues (9), Organosulfur compounds (7), Lignans, neolignans and related compounds (6), and Other (51) (**Figure 7B**). Principal component analysis (PCA) analysis showed a distinct separation between the samples from waxy and glossy plants, indicating differences in metabolite abundance under natural drought conditions (**Figure 7C**).

VLCFAs and derivatives pool of cuticular wax analysis showed that aldehyde (C22, C23), alcohols (C20), and esters (C29) were significantly increased in glossy plants under drought (**Figure 7D**). Meanwhile, it was found that free fatty acid (C16-C18) has no differences between waxy and glossy plants (**Supplementary Figure 14**). The precursors for wax synthesis in higher plants are primarily saturated fatty acids, including palmitic acid (C16:0) and stearic acid (C18:0), as well as unsaturated fatty acids such as oleic acid (C18:1), linoleic acid (C18:2), and α-linolenic acid (C18:3). The synthetic pathway is influenced not only by drought but also induced by the plant hormones abscisic acid (ABA) and jasmonic acid (JA) (Zhang et al., 2025). Metabolomics analysis revealed that the relative intensity of (+/-)-dihydrojasmonic acid and alpha-eleostearic acid in glossy rapeseed under drought stress were significant difference, while ABA, palmitic acid, 3-keto palmitic acid, stearic acid, and DL-7-hydroxy stearic acid were no changes (**Figure 7E**, **S7**). KEGG enrichment analysis founded that DEMs involved in the pathways of amino acid metabolism and biosynthesis (map00470, map01230, map00480), arginine and proline metabolism (map00330), pentose phosphate (map00030), tropane, piperidine and pyridine alkaloid biosynthesis (map00960), linoleic acid metabolism (map00591, map00592), ABC transporters (map02010), pantothenate and CoA biosynthesis (map00770), 2-Oxocarboxylic acid metabolism (map00591), flavonoid biosynthesis (map00941), and glucosinolate biosynthesis (map00966) were significantly alter in glossy plants compared to waxy plants (**Figure 7F, G**). Most of DEMs involved in these enrichment pathways were compatible osmolytes in plants under drought stress, including proline, betaine, soluble sugars (sucrose, glucose and trehalose), and polyols. These substances jointly alleviate drought-induced damage by reducing cellular water potential, maintaining cell turgor, stabilizing the structure of biomacromolecules and scavenging reactive oxygen substances. The majority of DEMs identified in these enriched pathways function as compatible osmolytes that support plants during drought stress. These include proline, betaine, soluble sugars (such as sucrose, glucose, and trehalose), and polyols. These compounds collectively alleviate drought-induced damage by decreasing cellular water potential, preserving cell turgor, maintaining the stability of biomacromolecules, and scavenging reactive oxygen species. However, a significant downregulation of most of these DEMs was observed in glossy plants (**Figure 7G**), potentially contributing to the increased drought sensitivity of glossy plants.

### 3.6 Integrated transcriptome–proteome–metabolome analysis

**Figure 8:**
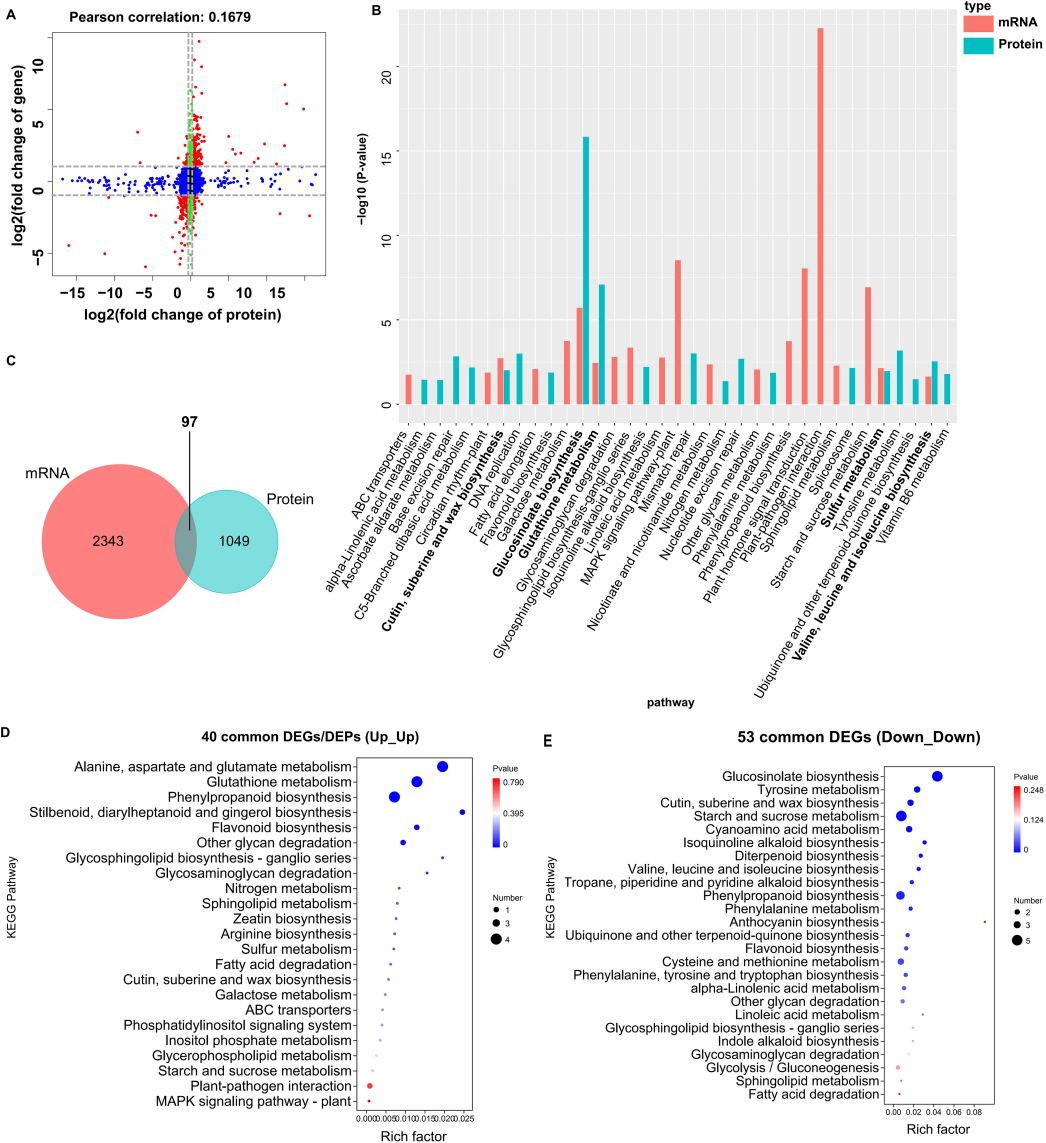
Transcriptome-proteome integration uncovers the common DEGs/DEPs under drought stress in glossy plants. **(A)** Nine-quadrant plot illustrating the correlation analysis between proteins and genes. The horizontal and vertical axes represent fold changes in expression for proteins and genes, respectively. The horizontal and vertical dashed lines respectively represent the screening threshold lines at the transcriptional level (|log_2_FC| =1) and the protein level (|log_2_FC| =0.263). Red dot indicates significant differential expression at both the transcript and protein levels. Blue dot indicates significant differential expression only at the protein level. Green dot indicates significant differential expression only at the transcript level, and black dot indicates no significant differential expression at either level. **(B)** Merged pathways in transcriptomic and proteomic data. **(C)** Venn diagram showing the overlap between differentially expressed proteins and transcripts with their corresponding relationships. **(D, E)** KEGG pathway enrichment analysis of 40 common upregulated **(D)** and 53 common downregulated **(E)** DEMs/DEPs under natural drought conditions.

To better understand the molecular mechanism of glossy rapeseed response to drought stress, firstly, we performed an integrated analysis of transcriptomics and proteomics data by Pearson correlation. A nine-quadrant scatterplot association analysis showed that a total of 13,437 genes commonly detected at both the transcript and protein levels (**Figure 8A**, **Excel S1**). 393 genes/proteins were significantly associated between transcriptome and proteome based on the screening threshold line of transcriptome was set as |log2FC|>1 and *p* < 0.05 and the proteome was set as | log2FC|>0.263 and *p* < 0.05 (**Figure 8A**, **Excel S1**). Out of the 393 genes/proteins, 329 exhibited similar expression patterns, including 186 up-regulated (Quadrant 3), 143 down-regulated (Quadrant 7), while 64 were opposite expression patterns (Quadrant 1 and Quadrant 9) (Excel S1). Pathway enrichment analyses were conducted separately for the transcriptome and proteome. Five common pathways were identified among the top 20 pathways in both proteomic and transcriptomic analyses, including cutin, suberin, and wax biosynthesis, glucosinolate biosynthesis, glutathione metabolism, sulfur metabolism, and valine, leucine, and isoleucine biosynthesis (**Figure 8B**). Based on the quantitative and differential analyses of proteomics and transcriptomics, the overall correspondence between proteins and transcripts were obtained. A total of 97 common DEGs/DEPs were found (**Figure 8C**). Among these, 40 DEGs/DEPs distributed in Quadrant 3 displaying concordant increases, while 53 DEGs/DEPs distributed in Quadrant 7 exhibiting concordant decreases, and 4 DEGs/DEPs displaying opposing patterns (Excel S1). This result revealed a highly consistent relationship between the upregulation and downregulation of proteins and genes. Two *CER1, BnaA09G0721400ZS* and *BnaA09G0698500ZS*, belong to the down-regulated groups, which are the top 20 most significant changes among common DEGs/DEPs (**Supplementary Figure 15**). Therefore, the glossy plants suffer from a coordinated, multi-level suppression of core wax biosynthesis machinery. KEGG enrichment analysis revealed that 40 up-regulated DEGs/DEPs were enriched in pathways including alanine, aspartate and glutamate metabolism, glutathione metabolism, phenylpropanoid biosynthesis, flavonoid biosynthesis, ABC transporters, and MAPK signaling pathway (**Figure 8D**), where 53 down-regulated DEGs/DEPs were enriched pathways such as glucosinolate biosynthesis, cutin, suberine and wax biosynthesis, starch and sucrose metabolism, valine, leucine and isoleucine biosynthesis, and anthocyanin biosynthesis (**Figure 8E**). These metabolic pathways are the core functional pathways driven by transcription-protein synergy for cuticular wax biosynthesis and response to the drought stress in rapeseed.

**Figure 9:**
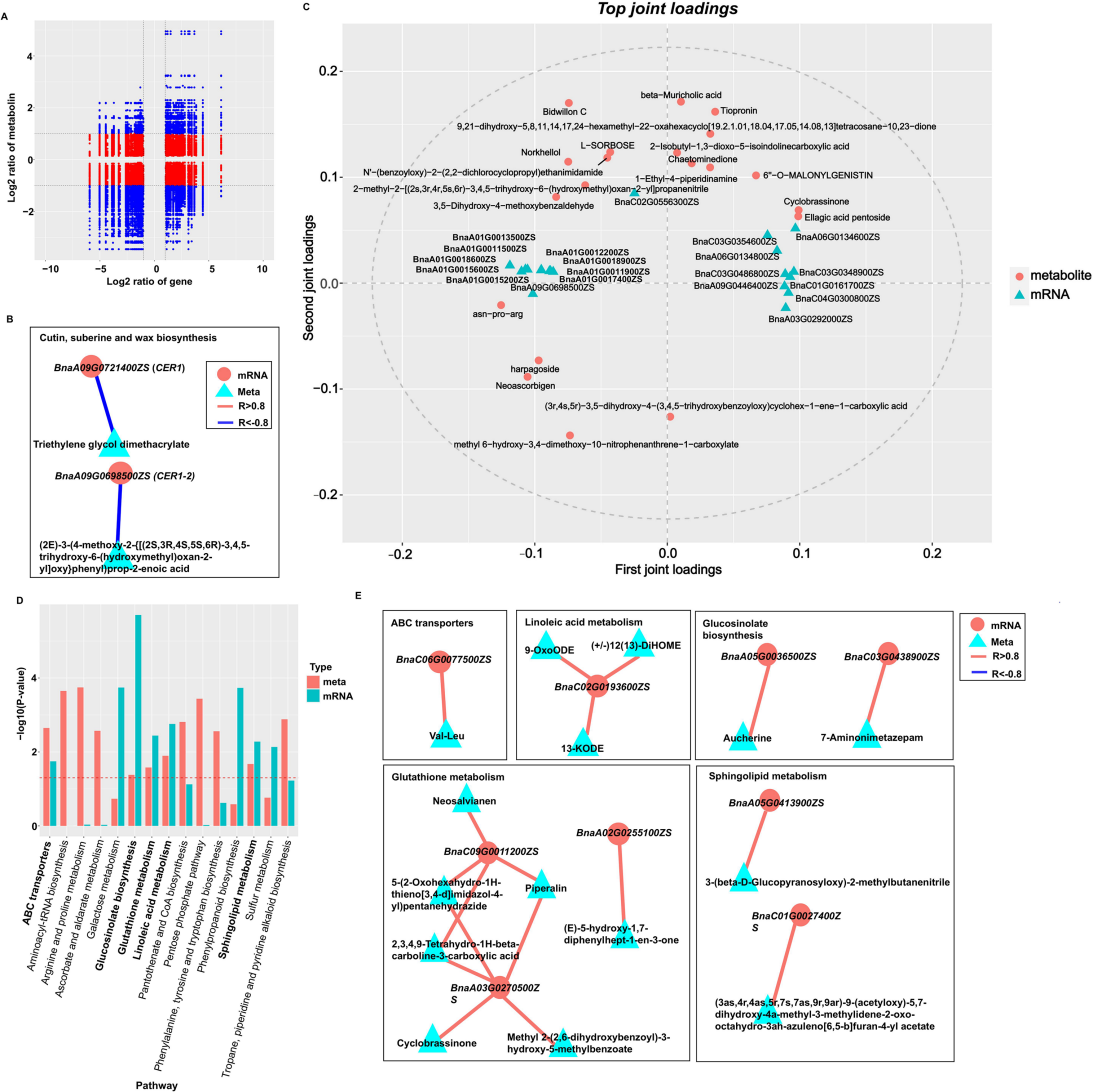
Combined 97 common DEGs/DEPs and metabolome analysis. **(A)** Nine-quadrant plot analysis the correlation between DEGs/DEPs and DEMs. **(B)** Analysis of correlation network diagram of wax biosynthesis pathways Two *CER1* genes, *BnaA09G0721400ZS* and *BnaA09G0698500ZS*, were significantly negatively correlated with triethylene glycol dimethacrylate (TEGDMA) and (2E)-3-(4-methoxy-2-{[(2S,3R,4S,5S,6R)-3,4,5-trihydroxy-6-(hydroxymethyl)oxan-2-yl]oxy}phenyl)prop-2-enoic acid, respectively. **(C)** O2PLS analysis plots. The top20 significantly correlated DEGs and DEMs in the transcriptome and metabolome in loadings plot. **(D)** KEGG pathway enrichment analysis of metabolites and related transcripts. The red dashed line indicates the threshold at *p* = 0.05, and items above this line represent results with *p* < 0.05. **(E)** Five common pathways correlation network analysis.

To comprehensively elucidate the intrinsic molecular characteristics of glossy rapeseed plants response to drought stress, Pearson correction, O2PLS, and KEGG pathway enrichment analysis were employed to integrate transcriptome, proteome, and metabolome data. To assess the correlation between 97 DEGs/DEPs and 1,078 DEMs in glossy plants subjected to drought stress, a nine-quadrant plot was employed to depict the distribution of the differences in multiple DEMs with a |correlation coefficient|>0.8 to DEGs and a correlation test *p* value< 0.05 in each group (**Figure 9A**). The correlation analysis revealed that 6, 615 DEGs were positively correlated to 385 DEMs in Quadrant 3 and Quadrant 7 (Excel S1), where the DEGs and DEMs exhibiting concordant increases in Quadrant 3 and 1,187 DEGs positively correlated to 100 DEMs (**Figure 9A**, **Excel S1**). In Quadrant 7, the DEGs and DEMs exhibiting concordant decreases, where 5,428 DEGs positively correlated to 285 DEMs (**Figure 9B**, **Excel S1**). In addition, 3521 DEGs were negatively correlated to 331 DEMs in Quadrant 1 and Quadrant 9 (**Figure 9A**, **Excel S1**). Further screening the top 100 items with the smallest *p*-values for correlation network analysis (**Supplementary Figure 16**, **Excel S1**), which including 70 positive correlation and 30 negative correlation DEG_DEM pairs. The 70 positive pairs significantly enriched in other glycan degradation, glutathione metabolism, glucosinolate biosynthesis, sphingolipid metabolism, flavonoid biosynthesis, galactose metabolism, and alanine, aspartate and glutamate metabolism (**Supplementary Figure 17A**). The 30 negative pairs significantly enriched in cutin, suberine and wax biosynthesis, flavonoid biosynthesis, glycosphingolipid biosynthesis, phenylpropanoid biosynthesis, glycosaminoglycan degradation, and stilbenoid, diarylheptanoid and gingerol biosynthesis (**Supplementary Figure 17B**). As we excepted, *BnaA09G0721400ZS* (*CER1*) and the paralog *BnaA09G0698500ZS* (*CER1-2*) in 30 negatively correlation group of the top 100 correlation were involved in cutin, suberine and wax biosynthesis and significantly correlated with triethylene glycol dimethacrylate (TEGDMA) and (2E)-3-(4-methoxy-2-{[(2S,3R,4S,5S,6R)-3,4,5-trihydroxy-6-(hydroxymethyl) oxan-2-yl] oxy} phenyl) prop-2-enoic acid, respectively (**Figure 9B**).

The O2PLS model was adopted to assess the overall association between 97 common DEGs/DEPs and metabolome to identify hub genes and metabolites that significantly contribute to the inter-omics regulatory network. The results showed that 20 DEGs and DAMs with the strongest associations were screened out (**Figure 9C**), which shows that the top 20 DEGs with the strongest influence on metabolomic were *BnaA01G0018600ZS* (*MES9*), *BnaA01G0015600ZS* (*MEE59*), *BnaA06G0134600ZS* (*PR5*), *BnaA01G0013500ZS* (*FMDA*), *BnaA01G0011500ZS* (*PCK1*), *BnaA09G0698500ZS* (*CER1*), *BnaC03G0348900ZS* (*Ukown*), *BnaA01G0015200ZS* (*CYP81D1*), *BnaA01G0017400ZS* (*CLASRP*), *BnaC01G0161700ZS* (*TLP1*), *BnaA03G0292000ZS* (*Ukown*), *BnaC04G0300800ZS* (*BGL*), *BnaA01G0012200ZS* (*HAT22*), *BnaC03G0486800ZS* (*PR1*), *BnaA01G0018900ZS* (*HHO5*), *BnaA09G0446400ZS* (Ukown), *BnaC02G0556300ZS* (*RAB18*), *BnaA06G0134800ZS* (*PR5*), *BnaC03G0354600ZS* (*PR4B*), *BnaA01G0011900ZS* (*COX6A*). In addition, *BnaA09G0721400ZS* (*CER1*) also as the top 35 DEGs strongest influence on metabolomic also was identified. The top 20 DAMs with high impact on transcriptomic were Bidwillon C (MN12018), beta-Muricholic acid (MN17566), Tiopronin (MP436), methyl 6-hydroxy-3,4-dimethoxy-10-nitrophenanthrene-1-carboxylate (MP16561), 9,21-dihydroxy-5,8,11,14,17,24-hexamethyl-22-oxahexacyclo [19.2.1.01,18.04,17.05,14.08,13] tetracosane-10,23-dione (MN18323), Neoascorbigen (MN12641), Norkhellol (MN8133), L-SORBOSE (MN4348), asn-pro-arg (MP18061), N’-(benzoyloxy)-2-(2,2-dichlorocyclopropyl) ethanimidamide (MP12517), (3r,4s,5r)-3,5-dihydroxy-4-(3,4,5-trihydroxybenzoyloxy) cyclohex-1-ene-1-carboxylic acid (MN12262), 2-Isobutyl-1,3-dioxo-5-isoindolinecarboxylic acid (MP7140), 6’’-O-MALONYLGENISTIN (MP24434), Harpagoside (MP24341), Cyclobrassinone (MN8132), Ellagic acid pentoside (MN16690), 3,5-Dihydroxy-4-methoxybenzaldehyde (MP5747), Chaetominedione (MP13731), 1-Ethyl-4-piperidinamine (MP3326), 2-methyl-2-[(2s,3r,4r,5s,6r)-3,4,5-trihydroxy-6-(hydroxymethyl) oxan-2-yl] propanenitrile (MP9510). They were all isoflavonoids, amino acids and derivatives, carbohydrates and carbohydrate conjugates, prenol lipids, and others class metabolites, which proved that these 20 DEGs and 20 DAMs were closely related to the drought response of glossy rapeseed.

Finally, the common pathways of the two omics difference enrichment analyses were conducted. The results uncovered that14 distinct pathways were co-enriched in multi-omics approach, including ABC transporters, arginine and proline metabolism, ascorbate and aldarate metabolism, glucosinolate biosynthesis, glutathione metabolism, pantothenate and CoA biosynthesis, linoleic acid metabolism, phenylpropanoid biosynthesis, and sphingolipid metabolism (**Figure 9D**). Among these, five pathways were significantly co-enriched in ABC transporters, glucosinolate biosynthesis, glutathione metabolism, linoleic acid metabolism, and sphingolipid metabolism with p<0.05 (**Figures 9D, E**). DEGs/DEPs and DEMs involved in these five pathways all exhibited significantly positively correlation (**Figure 9E**). For example, *BnaC06G0077500ZS* was significantly positively correlation with Val-Leu in ABC transporters pathway, *BnaC02G0193600ZS* was significantly positively correlation with 9-OxoODE, (+/-)12(13)-DiHOME, and 13-KODE in linoleic acid metabolism pathway, and *BnaC09G0011200ZS*, *BnaA03G0270500ZS*, and *BnaA02G0255100ZS were* positively correlation with seven metabolites in glutathione metabolism (**Figure 9E**). These findings in multi-omics analysis indicated that the glossy plants’ response to drought is not limited to wax deficiency but involves broad metabolic reprogramming.

## 4 Discussion

### 4.1 *CER1* as the key candidate gene for leaf cuticular wax biosynthesis and drought response in rapeseed

In this study, the dominant glossy mutant *‘hy7201’*, characterized by reduced total wax content, altered composition, decreased crystal density, and increased cuticle permeability, these physiological and wax changes directly explain the glossy appearance and likely contribute to drought sensitivity (**Figure 1**). Furthermore, *BnaA09G0721400ZS* (*CER1*) was identified as the key candidate gene controlling leaf cuticular wax and responding to drought, as reported in previous studies (Pu et al., 2013; Wang et al., 2020; Zhang et al., 2025), through BSA-seq, sequence analysis, and a multi-omics approach in the current study. Interestingly, both CER1 and its paralog ‘CER1-2’ were significantly downregulated at both transcription and protein levels under drought stress. Unlike single-gene suppression, the synchronous repression of two functionally redundant homologous enzymes creates a cumulative inhibitory effect on the core wax biosynthetic pathway at transcriptional and post-transcriptional levels. Such coordinated multi-level suppression severely blocks the synthesis of very-long-chain alkanes and other major wax components, resulting in sparse wax crystal deposition, reduced wax content and impaired cuticle barrier function (Chaudhary et al., 2021; Li et al., 2025a; Li-Beisson et al., 2013; Pascal et al., 2019; Schirmer et al., 2010). These structural defects further aggravate non-stomatal water loss and drought sensitivity in the mutant. Therefore, the glossy mutant/plant in this study suffers from a coordinated, multi-level suppression of core wax biosynthesis machinery and exhibits more vulnerable to drought stress.

### 4.2 Intrinsic molecular characteristics of glossy plants under drought in rapeseed

**Figure 10:**
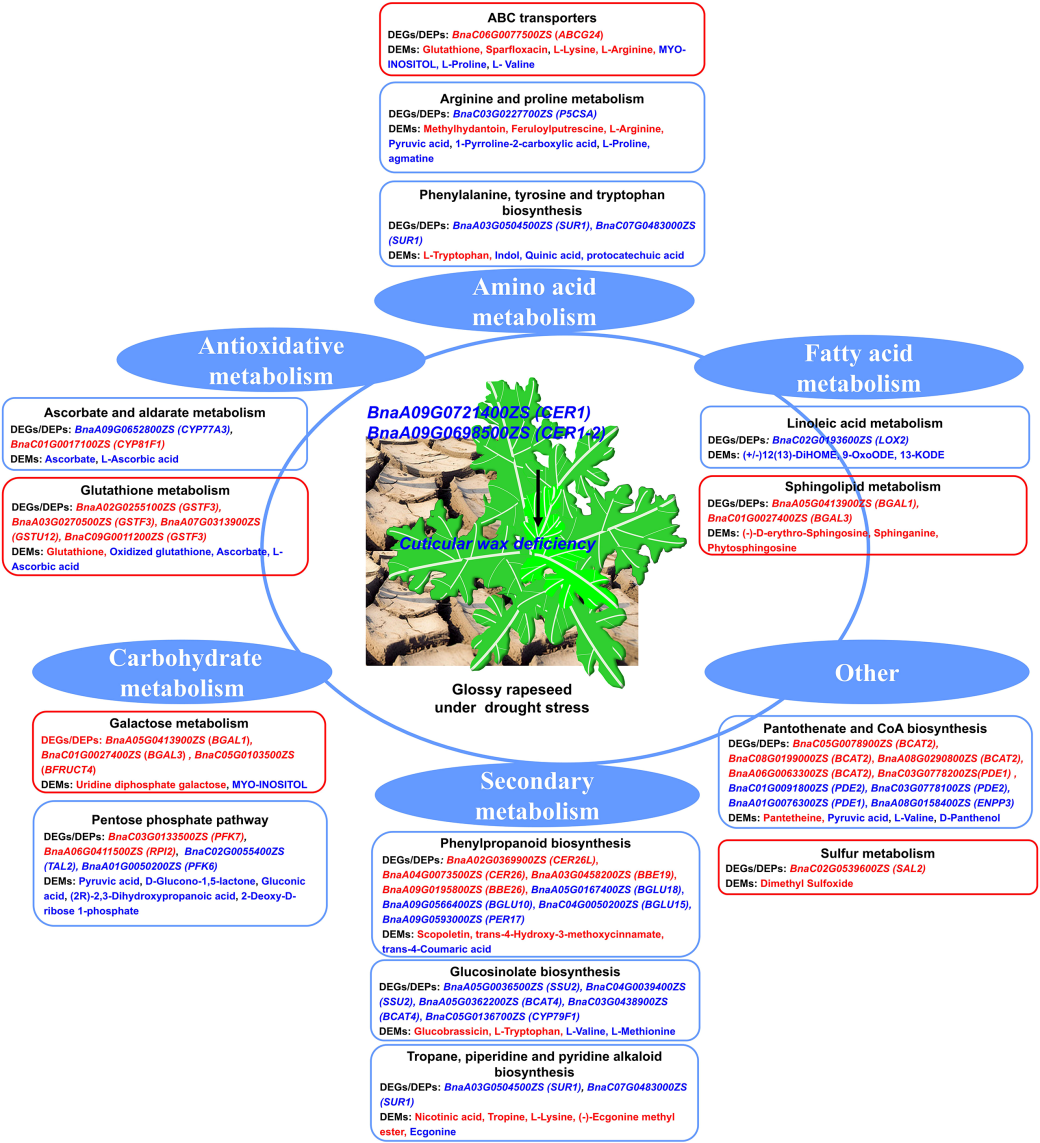
DEGs, DEMs, DAMs, and pathways for drought tolerance in glossy plants. Red font indicates upregulated DEGs, DEMs, and DAMs, while blue font indicates downregulated DEGs, DEMs, and DAMs. The red box highlights upregulated pathways, and the blue box highlights downregulated pathways.

In plants, morphology and physiological processes are regulated by genes, proteins, and metabolites. Metabolites serve as indicators of gene expression and regulatory activities, resulting in diverse phenotypes when plants encounter abiotic stresses (Liu et al., 2021; Roy et al., 2026; Zhang et al., 2025). In the present study, the simultaneous repression of two functionally redundant homologous enzymes (CER1 and CER1-2) produces a cumulative inhibitory effect on the core wax biosynthetic pathway, severely blocking the synthesis of very-long-chain alkanes and other major wax components. This leads to a significant reduction in alkanes (C29, C34, C43) and ketones (C29, C30), while fatty acids (C31) and aldehydes (C23, C25, C32) accumulate significantly in the glossy mutant ‘hy7201’ (**Figures 1F, G**). In the absence of core wax synthesis via the alkane-forming pathway, wax components are reallocated, resulting in the accumulation of alcohols and wax esters in response to drought stress (**Figure 7D**). Meanwhile, multi-omics joint analysis revealed that the glossy mutant under drought stress is enriched in 14 distinct metabolic pathways, primarily involving fatty acid metabolism, amino acid metabolism, secondary metabolism, antioxidative metabolism, and carbohydrate metabolism (**Figures 9D**, **10**). Consequently, the glossy mutant’s response to drought is not limited to wax deficiency but involves broad metabolic reprogramming.

The gene/protein-metabolite correlation matrix and scatter plots to explicitly display the negative correlation trends between suppressed CER1/CER1–2 and accumulated metabolites. *CER1* and *CER1–2* display significant negative correlation with triethylene glycol dimethacrylate (TEGDMA) and (2E)-3-(4-methoxy-2-{[(2S,3R,4S,5S,6R)-3,4,5-trihydroxy-6-(hydroxymethyl) oxan-2-yl] oxy} phenyl) prop-2-enoic acid, respectively (**Figure 9B**). TEGDMA may be involved in the mitochondrial damage pathway by inhibiting respiratory chain complex I, leading to increased ROS accumulation, decreased ATP content, and collapse of mitochondrial membrane potential, ultimately resulting in cell death (Qader, 2026), whereas (2E)-3-(4-methoxy-2-{[(2S,3R,4S,5S,6R)-3,4,5-trihydroxy-6-(hydroxymethyl) oxan-2-yl] oxy} phenyl) prop-2-enoic acid involved in the phenylpropanoid metabolic pathway and shares upstream carbon metabolic flux with the pathway of cutin, suberin and wax biosynthesis (Ninkuu et al., 2025). Therefore, the accumulation of TEGDMA inflect the oxidative damage in glossy plants under drought stress, whereas the upregulation of phenylpropanoids as antioxidant compensation (Šamec et al., 2021; Yin et al., 2025; Zagoskina et al., 2023). Meanwhile, phenylpropanoid metabolic pathway shares upstream carbon metabolic flux with the pathway of cutin, suberin and wax biosynthesis, which realize the flow of wax precursor substances into phenylpropanoid pathway (Ullah et al., 2022; Ninkuu et al., 2025). Additionally, the block of core wax synthesis in the alkane-forming pathway appears led to the activation of the alkane-forming pathway, where alcohols and wax esters are accumulated in glossy plant under drought stress (**Figure 7D**). Furthermore, the sphingolipid metabolism was also significantly upregulated in glossy plants under drought stress, and which shares a common precursor, VLCFA-CoA, with the wax synthesis pathway (Liu et al., 2026b). Accumulated sphingolipids, as critical components of the plant plasma membrane and endomembrane system, contribute to membrane fluidity and biophysical order, thereby enhancing plant defense (Ali et al., 2025; Huby et al., 2020). Therefore, the absence of alkane-forming pathway leading to the wax precursors, such as VLCFA-CoA, were reallocated to wax synthesis alcohols-forming pathway, phenylpropanoid metabolism pathway, and sphingolipid metabolism pathway to regulate the VLCFA pool. Consequently, the accumulation or depletion of specific wax-related metabolites likely reflects disrupted substrate conversion rather than passive leakage.

Additionally, in our study, proline, valine, and methionine were significantly reduced in glossy plants, whereas tryptophan, lysine, and arginine were upregulated (**Figure 10**). Proline, a key AA and osmoprotectant, was significantly downregulated in glossy plants under drought conditions leading to redox imbalance (GSH/GSSG and NADPH/NADP+) (Bowne et al., 2012; Raza et al., 2026). Additionally, proline and valine have been reported to serve as complementary energy sources and provide substrates for ATP synthesis (Hildebrandt et al., 2015). L-ascorbic acid (AsA), another important antioxidant that plays a critical role in plant responses to drought (Yang et al., 2022b; Raza et al., 2026), was significantly reduced in glossy plants under drought in this study. Therefore, the upregulation of glutathione (GSH) metabolism and galactose metabolism might as compensatory stress-response mechanisms for proline and valine downregulation to maintain ROS and energy balance. In addition, GSH also involved in the detoxification of methylglyoxal, the formation of phytochelatins, interactions with plant hormones and other signaling molecules, and its redox state triggers signal transduction (Ahmad et al., 2016; Chali et al., 2026). Furthermore, linoleic acid is known to be oxidized during stress responses, leading to the synthesis of important signaling molecules such as jasmonic acid, which participate in multiple signaling pathways (Savchenko et al., 2014). In the current study, dihydrojasmonic acid (DHJA) in the jasmonic acid biosynthesis pathway was upregulated, speculating linoleic acid may be further desaturated to form alpha-linolenic acid and DHJA response to drought. Glucobrassicin, a type of glucosinolate, was significantly upregulated and plays roles in water conservation by inducing stomatal closure and contributing to osmotic adjustment under drought conditions (Nicolas-Espinosa et al., 2023; Salehin et al., 2019; Shawon et al., 2020; Ye et al., 2020). Therefore, the changes such as glucosinolate, and linoleic acid as part of a feedback-loop regulation, where the plant attempts to compensate for the disrupted cuticular barrier by activating other defense and osmotic adjustment pathways.

Under drought stress, starch and sucrose metabolism serves as a crucial pathway for sugar metabolism, mediating the synthesis of osmotic adjustment substances such as soluble sugars, eliminating reactive oxygen species, and functioning as signaling molecules to initiate defense responses (Wang et al., 2024; Guo et al., 2026). However, this study found that galactose metabolism and the pentose phosphate pathway, rather than starch and sucrose metabolism, were significantly enriched in glossy plants under drought conditions. These findings indicate that galactose metabolism and the pentose phosphate pathway are the primary sugar metabolism pathways in glossy rapeseed during drought. Three genes, *BnaA05G0413900ZS* (*BGAL1*), *BnaC01G0027400ZS* (*BGAL3*), and *BnaC05G0103500ZS* (*BFRUCT4*) were significantly upregulated in galactose metabolism. Notably, BGAL1 and BGAL3 participate in cell wall remodeling and facilitate the accumulation of soluble sugars under drought conditions (Kriechbaum et al., 2020; Liu et al., 2020; Yang et al., 2021). Additionally, uridine diphosphate galactose (UDP-Gal) was markedly elevated in glossy plants, influencing the flow direction in raffinose to generate osmotic protective substances such as melibiose, D-galactose, and D-glucose. Furthermore, UDP-Gal is involved in glycan glycosylation modifications, enabling the attachment of β-D-galactose to the termini of glycoprotein N/O polysaccharides, arabinogalactan proteins (AGPs), and pectin polysaccharides (Ge et al., 2025). BnaC03G0133500ZS (PFK7) and BnaA01G0050200ZS (PFK6) are involved in the pentose phosphate pathway, facilitating the conversion of fructose-6-P to fructose-1,6-P (Kruger and von Schaewen, 2003). These genes were up-regulated and down-regulated, respectively, in glossy rapeseed under drought conditions. Furthermore, BnaA06G0411500ZS (RPI2) and BnaC02G0055400ZS (TAL2), which serve as key enzymes in the non-oxidative stage of the pentose phosphate pathway, also exhibited significant up-regulation and down-regulation in glossy rapeseed under drought, respectively. Finally, the end products of glycolysis and energy supply from the TCA cycle, including pyruvic acid and intermediate carbon metabolites such as (2R)-2,3-Dihydroxypropanoic acid, along with the products of the PPP pathway, D-glucono-1,5-lactone, gluconic acid, and 2-Deoxy-D-ribose 1-phosphate, were all significantly down-regulated. Under drought stress, the PPP serves as a crucial respiratory pathway that compensates for energy and NADH deficiencies in the Embden-Meyerhof-Parnas pathway–tricarboxylic acid cycle (EMP–TCA) (Hu et al., 2021, 2022b).

Collectively, glossy rapeseeds signify a highly dynamic and complex transcriptomic, proteomics, and metabolic reprogramming to establish a unique homeostasis under drought conditions (**Figure 10**). The core mechanism involves: (1) regulation of VLCFA pool through upregulation of sphingolipid metabolism to enhance membrane fluidity and biophysical order; (2) significant enhancement of enzyme systems related to glutathione metabolism for antioxidant defense; (3) augmentation of epidermal defense functions coupled with galactose accumulation to facilitate osmotic regulation and energy supply; (4) the modulation of interaction among amino acids, plant hormones, and secondary metabolism.

### 4.3 A “multi-pathway synergistic approach’’ for future breeding strategies

Understanding drought resistance mechanisms in rapeseed is critical for enhancing resilience to intensifying climate change. This study conducted a multi-omics analysis of waxy and glossy plants from the F_1_ generation of a self-cross population derived from the glossy mutant ‘*hy7201*’ under natural field drought conditions. Although a strictly well-watered control was not included, all materials were subjected to the same drought environment, effectively minimizing environmental interference. Furthermore, results obtained under field conditions, as opposed to controlled environments, align more closely with actual agricultural practices, offering practical implications for the breeding of drought-resistant wax traits in rapeseed. Drought stress will amplify specific molecular signatures between waxy and glossy plants. Natural drought stress significantly induces the differential expression of genes, proteins, and metabolic pathways not only associated with cuticular wax biosynthesis, but also involved in AA metabolism, secondary metabolism, carbohydrate metabolism, and antioxidative metabolism, thereby future research can simultaneously optimize wax biosynthesis and other drought-response metabolism for using a comprehensive “germplasm–gene–metabolite” breeding framework.

Leaf cuticular wax serves as a vital barrier for drought stress in rapeseed. Germplasms with a higher total cuticular wax content in their leaves exhibited greater drought tolerance (Long et al., 2023). Most glossy leaf mutants reported in *Brassicaceae* are controlled by recessive genes, which can only display the glossy phenotype in homozygous recessive plants. Their hybrid F_1_ progenies exhibit wild-type wax coverage, so glossy traits cannot be directly utilized in hybrid cultivars, and multiple selfing generations are required to screen homozygous glossy individuals (Han et al., 2021; Huang et al., 2025; Ji et al., 2021; Liu et al., 2021; Qi et al., 2025; Yang et al., 2022a). In contrast, the glossy leaf trait controlled by a single dominant gene in this study can be stably manifested in both heterozygous and homozygous plants. This feature brings unique breeding values that the F_1_ hybrids derived from crosses between homozygous dominant glossy lines and conventional waxy lines uniformly display glossy leaves, realizing the direct utilization of glossy traits in hybrid rapeseed varieties. The glossy leaf acts as a visible morphological marker to rapidly identify false hybrid seedlings at the seedling stage, greatly simplifying seed purity identification in seed production. Additionally, the glossy mutant *‘hy7201’* derived from the unique yellow-seed rapeseed background ‘HY7201’. The yellow-seeded trait is a desirable breeding characteristic in rapeseed that can greatly improve seed oil yield and quality (Qu et al., 2023). Develop functional molecular markers closely linked to dominant glossy leaves and yellow seeds, carry out molecular marker-assisted drought resistance breeding, and precisely aggregate high-quality multi-resistance genes.

The regulatory mechanisms governing leaf cuticular wax synthesis and other metabolites in rapeseed under drought provides a series of related genes or transcription factors (TF). Thereby, we also highlight using genetic engineering tools to simultaneously optimize cuticular wax biosynthesis and other metabolism pathways. We can undergo overexpress genes like *CER1* to improve the cuticular wax*, BGAL1* and *BGAL3* to promote the accumulation of galactose and sphingolipids enhancing drought tolerance. Meanwhile, CRISPR/Cas technology also provides a precise and efficient tool for multiplex editing of metabolic networks (Li et al., 2025b; Hao et al., 2026). This approach provides “full gene package” and could simultaneously optimize multiple biosynthetic or regulatory genes (Raza et al., 2025). By identifying and expressing the common TF in upstream of these drought-related metabolic pathway genes, drought tolerance can be jointly improved through multiple pathways by co-expressing genes and metabolites, for example MYBs (Jin et al., 2025; Wu et al., 2026). Therefore, the drought tolerance of rapeseed is precisely enhanced from the simultaneous response of multiple genes to multiple metabolites.

## 5 Conclusion

In summary, we identified a dominant glossy mutant *hy7201* in rapeseed with reduced cuticular wax, altered composition, and increased cuticle permeability. BSA-seq pinpointed *BnaA09G0721400ZS* (CER1) as key candidate genes. Under natural drought stress, integrated multi-omics analysis revealed that both *CER1* and its paralog ‘*CER1-2*’ were significantly downregulated at the transcript and protein levels. Consequently, we confirmed our hypothesis that the glossy mutation suppresses wax biosynthesis at multiple molecular layers. Furthermore, significant negative correlations between *CER1* expression and differentially expressed metabolites support a causal role for *CER1* in wax metabolite imbalance. The multi-omics approach also uncovered 14 enriched pathways, including cutin/suberin/wax biosynthesis, ABC transporters, glutathione metabolism, linoleic acid metabolism, and glucosinolate metabolism. Therefore, the glossy mutant’s drought response involves not only a defective cuticular barrier but also broad metabolic and signaling reprogramming.

## Data Availability

The BSA-seq and RNA-seq data presented in the study are deposited in the Sequence Read Archive (SRA) database of the National Center for Biotechnology Information (NCBI) repository, accession number PRJNA1467310. The mass spectrometry proteomics data have been deposited to the ProteomeXchange Consortium (https://proteomecentral.proteomexchange.org) via the iProX partner repository with the dataset identifier PXD081103. The metabolomics data have been deposited to MetaboLights repository with the study identifier MTBLS15048.
