# Supplementary material for: Integrated multi-omics analysis reveals candidate genes for cuticular wax biosynthesis and molecular characteristics of a glossy mutant in rapeseed under natural drought stress
Source: Front Plant Sci. 2026 Jul 29;17:1874927. doi: 10.3389/fpls.2026.1874927 (PMC13463271; doi:10.3389/fpls.2026.1874927)
Supplement: Supplementary file 1 [file DataSheet1.docx]

Supplementary Material

Integrated multi-omics analysis reveals candidate genes for cuticular wax biosynthesis and molecular characteristics of a glossy mutant in rapeseed under natural drought stress

Lei Lei, Xianmin Meng, Weirong Wang, Hongwei Li, Jifeng Zhu^*^

Key Laboratory of Germplasm Innovation and Genetic Improvement of Grain and Oil Crops (Co-construction by Ministry and Province), Ministry of Agriculture and Rural Affairs, Key Laboratory of Agricultural Genetics and Breeding of Shanghai, Crop Breeding and Cultivation Research Institute, Shanghai Academy of Agricultural Sciences, Shanghai, China

*** Correspondence:**
Jifeng Zhu
[zhujifeng@saas.sh.cn](mailto:zhujifeng@saas.sh.cn)

**Supplementary Figure 1. Meteorological data from November to December 2025 and the natural drought conditions in this study.** (A) Soil moisture content in the 1–28 cm soil layer from November to December 2025. (B) Daily rainfall during November to December 2025. (C) Maximum, minimum, and average daily air temperatures during November to December 2025. The dotted lines in the figure indicate the drought stress period (November 14 to 30, 2025) in this study. Meteorological data were obtained from the XIHE-ENERGY platform (https://xihe-energy.com/).

**Supplementary Figure 2. Alignment of promoter sequences of *BnaA09G0721400ZS* (*CER1*) in the reference genome ‘ZS11’, the wild type ‘HY7201’, and the waxy and glossy lines.**

**Supplementary Figure 3. Alignment of genomic sequences of *BnaA09G0721400ZS* (*CER1*) in the reference genome ‘ZS11’, the wild type ‘HY7201’, and the waxy and glossy lines.**

**Supplementary Figure 4.** **CDS sequences of *BnaA09G0721400ZS* (*CER1*) in the reference genome ‘ZS11’, the wild type ‘HY7201’, and waxy and glossy lines.**

**Supplementary Figure 5. Multiple sequence alignment of the putative protein sequences of BnaA09G0721400ZS (CER1) from the reference genome ‘ZS11’, the wild type ‘HY7201’, and the waxy and glossy lines.**

**Supplementary Figure 6. Alignment of promoter sequences of *BnaA09G0714600ZS* (*KCS1*) in the reference genome ‘ZS11’, the wild type ‘HY7201’, and the waxy and glossy lines.**

**Supplementary Figure 7. CDS/genomic sequences of *BnaA09G0714600ZS* (*KCS1*) in the reference genome ‘ZS11’, the wild type ‘HY7201’, and waxy and glossy lines.**
 **Supplementary Figure 8. Multiple sequence alignment of the putative protein sequences of BnaA09G0714600ZS (KCS1) from the reference genome ‘ZS11’, the wild type ‘HY7201’, and the waxy and glossy lines.**

**Supplementary Figure 9.** **GO term analysis of 2,327 DEGs between the waxy and glossy plants under natural drought conditions**

**Supplementary Figure 10.** **GO term and KEGG pathway analysis of upregulated and downregulated DEGs between waxy and glossy plants under natural drought conditions.** The top 20 GO term enrichments of upregulated DEGs (A) and downregulated DEGs (B). The top 20 of KEGG pathway enrichments of upregulated DEGs (C) and downregulated DEGs (D).

**Supplementary Figure 11.** **Radar chart of the top 40 differentially expressed proteins (DEPs) between waxy and glossy plants under natural drought conditions.** The position of the candidate protein BnaA09G0721400ZS(CER1) is marked with a blue asterisk

**Supplementary Figure 12.** **GO term and KEGG pathway enrichment analysis of upregulated and downregulated DEPs.** (A) GO term enrichment of upregulated DEPs in glossy plants. (B) GO term enrichment of downregulated DEPs in glossy plants. (C) KEGG pathway enrichment of upregulated DEPs in glossy plants. (D) KEGG pathway enrichment DEPs downregulated in glossy plants.

**Supplementary Figure 13.** **Domain enrichment analysis of differentially expressed proteins (DEPs) between waxy and glossy plants under natural drought conditions.**

**Supplementary Figure 14. The relative intensity of fatty acids and ABA between waxy and glossy plants under drought.**

**Supplementary Figure 15. Expression levels of the top 20 common differentially expressed genes at both the transcriptomic and proteomic levels.**

**Supplementary Figure 16. Correlation network analysis of the top 100 gene-metabolite pairs with the smallest *p*-values.** The color of the lines indicates the magnitude of the correlation coefficients, while the thickness of the lines represents the *p*-values. Node shapes differentiate genes from metabolites. The position marked by the blue circle highlights the location of *CER1* and its associated metabolites.

**Supplementary Figure 17. KEGG pathways enrichment analysis of 70 positive and 30 negative DEGs_DEMs pairs of the top 100 correlation network.** The color of the lines indicates the magnitude of the correlation coefficients, while the thickness of the lines represents the *p*-values. Node shapes differentiate genes from metabolites. The position marked by the blue circle highlights the location of *CER1* and its associated metabolites.

**Table S1 Primer sequences used for qRT-PCR analysis**

| **Gene name** | **ZS11 gene ID** | **Primer sequences (5'-3')** |
| --- | --- | --- |
| *BnaCER1* | *BnaA09G0721400ZS* | Forward: GGTTGGCTTCGTCATTCTA |
|  |  | Reverse: TGTATGTGCTATGGACCGC |
| *BnaCER1-2* | *BnaA09G0721400ZS* | Forward: CACACCCAATTCCGGACCA |
|  |  | Reverse: TATATGGACTCGGGCGTCGT |
| *BnaKCS1* | *BnaA09G0714600ZS* | Forward: TTAGGACCGATGGTTCTTCCTTTGT |
|  |  | Reverse: AACCGGTGCAAAGTCATTCTC |
| *BnaKCS1* | *BnaA09G0693100ZS* | Forward: AGCCGTTATGTTCGGTGCTT |
|  |  | Reverse: TGTTGGCGAGATCGATTGAGA |
| *BnaPAS2* | *BnaC09G0552800ZS* | Forward: GTACCAGGCACAGCCTCATT |
|  |  | Reverse: ACGCTGACCAAGCATGTACC |
| *BnaWSD6* | *BnaC01G0279200ZS* | Forward: GATGCGTTGGAGTTTATTGC |
|  |  | Reverse: CAAGGTATTGCGAGAGACCA |
| *BnaMAH1* | *BnaC09G0199200ZS* | Forward: CCGAAGTGGAGTTCGGTGAA |
|  |  | Reverse: AAGCCTCCACAAGATGACCG |
| *BnaMAH1* | *BnaC09G0199300ZS* | Forward: ACTGGTGACGACCCAATGTC |
|  |  | Reverse: CTTCTTCAGCGAAGTCGGCA |
| *BnaACTIN7* | *BnaC02G0037200ZS* | Forward: TATCCTCCGTCTCGATCTCGC |
|  |  | Reverse: CTTAGCCGTCTCCAGCTCTTG |

**Table S2 Primer sequences used for RT-PCR analysis**

| **Gene name** | **ZS11 gene ID** | **Primer sequences (5'-3')** |
| --- | --- | --- |
| *ProBnaCER1* | *BnaA09G0721400ZS* | Forward: TTGAGTTAGAGCAAGTGGACTG |
|  |  | Reverse: GTCTGATGATGAAGAGGGCT |
| *gBnaCER1* | *BnaA09G0721400ZS* | Forward: CGTGTCCTCTTCTAACTGGG |
|  |  | Reverse: TTATTGCTCTGCCTTGCC |
| *cBnaCER1* | *BnaA09G0721400ZS* | Forward: ggggcccataggcctgaattcATGGGACAAGATTACAGCTACAGCC |
|  |  | Reverse: tcacgcgtgactagtaagcttTCAGGGGTATTGGAAGTGATGTG |
| *ProKCS1* | *BnaA09G0714600ZS* | Forward: CACCACTTACGCTACACCCT |
|  |  | Reverse: GAATCCAAAGCACCGAAC |
| *gBnaKCS1* | *BnaA09G0714600ZS* | Forward: CCTTCTCCGAATCTGCCTAA |
|  |  | Reverse: GGTTTCCCTTATTGACTCCCT |
| *cBnaKCS1* | *BnaA09G0714600ZS* | Forward: ggggcccataggcctgaattcATGGAGAGAACAAACAGCATAGAGAT |
|  |  | Reverse:  tcacgcgtgactagtaagcttTCACTGCGTAATCTTAACCGGA |

**Table S3 The category, quantity and proportion of cuticular wax between ‘HY7201’ and ‘*hy7201’***

| **Category** | **HY7201** | | ***hy7201*** | |
| --- | --- | --- | --- | --- |
|  | **No.** | **Proportion (%)** | **No.** | **Proportion (%)** |
| Alkanes | 10 | 3.34 | 9 | 3.01 |
| Wax esters | 59 | 19.73 | 60 | 20.06 |
| Ketones | 9 | 3.01 | 9 | 3.01 |
| Aldehydes | 7 | 2.34 | 7 | 2.34 |
| Alcohols | 125 | 41.81 | 125 | 41.81 |
| Fatty acids | 21 | 7.02 | 21 | 7.02 |
| Amides | 8 | 2.68 | 9 | 3.01 |
| Terpenoids | 7 | 2.34 | 6 | 2.01 |
| Others | 53 | 17.73 | 53 | 17.73 |
| Total | 299 | 100 | 299 | 100 |
